# Distinct phenotype of neutrophil, monocyte, and eosinophil populations indicates altered myelopoiesis in a subset of patients with multiple myeloma

**DOI:** 10.3389/fonc.2022.1074779

**Published:** 2023-01-17

**Authors:** Krystle L. Ong, Marcus D. Davis, Kalyn K. Purnell, Hannah Cutshall, Harish C. Pal, Ashley N. Connelly, Christian X. Fay, Valeriya Kuznetsova, Elizabeth E. Brown, Zdenek Hel

**Affiliations:** ^1^Department of Pathology, University of Alabama at Birmingham, Birmingham, AL, United States; ^2^O’Neal Comprehensive Cancer Center, University of Alabama at Birmingham, Birmingham, AL, United States; ^3^Center for AIDS Research, University of Alabama at Birmingham, Birmingham, AL, United States

**Keywords:** multiple myeloma, myelopoiesis, cancer, granulocyte, neutrophil, monocyte, immune dysregulation

## Abstract

Hematologic malignancies, including multiple myeloma (MM), promote systemic immune dysregulation resulting in an alteration and increased plasticity of myeloid cell subsets. To determine the heterogeneity of the myeloid cell compartment in the peripheral blood of patients with MM, we performed a detailed investigation of the phenotype and function of myeloid subpopulations. We report that a subset of MM patients exhibits a specific myeloid cell phenotype indicative of altered myelopoiesis characterized by significant changes in the properties of circulating granulocytic, monocytic, and eosinophilic populations. The subset, referred to as MM2, is defined by a markedly elevated level of CD64 (FcγRI) on the surface of circulating neutrophils. Compared to healthy controls or MM1 patients displaying intermediate levels of CD64, neutrophils from MM2 patients exhibit a less differentiated phenotype, low levels of CD10 and CXC chemokine receptor 2 (CXCR2), increased capacity for the production of mitochondrial reactive oxygen species, and an expansion of CD16^neg^ immature neutrophil subset. Classical and patrolling monocytes from MM2 patients express elevated levels of CD64 and activation markers. MM2 eosinophils display lower levels of C-C Chemokine receptor 3 (CCR3), Toll-like receptor 4 (TLR4, CD284), and tissue factor (TF, CD142). The MM2 (CD64^high^) phenotype is independent of age, race, sex, and treatment type. Characteristic features of the MM2 (CD64^high^) phenotype are associated with myeloma-defining events including elevated involved/uninvolved immunoglobulin free light chain (FLC) ratio at diagnosis. Detailed characterization of the altered myeloid phenotype in multiple myeloma will likely facilitate the identification of patients with an increased risk of disease progression and open new avenues for the rational design of novel therapeutic approaches.

## Introduction

Multiple myeloma (MM) is a malignancy of post-germinal center terminally differentiated plasma cells producing antibodies and complexes of immunoglobulin heavy and light chains (1). Diagnosis is based on several myeloma-defining events including an accumulation of monoclonal plasma cells in the bone marrow microenvironment (BME), the presence of end-organ damage, or, in the absence of end-organ damage, elevated involved to uninvolved immunoglobulin free light chain (FLC) ratio and presence of at least one bone lesion by magnetic resonance imaging (1, 2). MM is associated with significant immune defects resulting in recurrent bacterial infections and other immune-related complications; however, the underlying causes of immune dysregulation in MM patients are not well understood (3–8). Immune reprograming in MM promotes angiogenesis and immunosuppression contributing to disease progression to extramedullary sites (9, 10).

Chronic inflammatory diseases, including solid and hematological malignancies, are frequently associated with pathologic dysregulation of the myeloid cell compartment including (11–19). During unresolved chronic inflammation, partial reprogramming of the BME results in altered granulopoiesis and recruitment of transcriptionally and physiologically distinct myeloid cell populations (12, 13, 20, 21). Recent studies demonstrate the plasticity of innate immune cells, specifically neutrophils, polymorphonuclear myeloid-derived suppressor cells (PMN-MDSCs), and monocyte subpopulations, in chronic pathologic conditions (12–19, 22–24).

Neutrophils represent the most abundant circulating leukocyte population equipped for the sensitive detection of bacterial and viral products during inflammatory responses (25). Previously, we demonstrated that neutrophils from individuals infected with human immunodeficiency virus-1 exert inhibitory effects on T-cell function and proliferation characterized by programmed death-ligand 1 (PD-L1) expression and release of reactive oxygen species (ROS) (23). PD-L1-expressing neutrophils play a critical role in the suppression of lymphocyte proliferation in endotoxemia (26). Reports of immunosuppressive properties of neutrophils and PMN-MDSCs, including increased arginase-1 expression and differences in phagocytic and oxidative burst capacities, support the critical role of neutrophils in fostering the tumor microenvironment promoting myelomagenesis (9, 17, 27). Neutrophils and PMN-MDSCs are expanded in the BME and peripheral blood of patients with MM (15, 28–32), protect MM cells from chemotherapy-induced toxicity resulting in reduced chemoselectivity (33), and inhibit T-cell immune response (34). Romano et al. described immunosuppressive high-density neutrophils in monoclonal gammopathy of undetermined significance (MGUS) and MM patients characterized by increased STAT3 and CD64 expression and hypothesized that this population may contribute to the increased susceptibility to infections and immune dysfunction supporting tumor progression (9). The neutrophil to lymphocyte ratio is increased in some MM patients and has been utilized to predict outcomes in newly diagnosed patients, patients that have previously undergone autologous hematopoietic stem cell transplant (aHSCT), and transplant-ineligible patients in MM (35–37).

Increased frequency of patrolling monocytes was observed in the BME of patients with MM relative to patients with pre-MM conditions, including MGUS and smoldering multiple myeloma (19). Patrolling monocytes promote an increase in osteoclast formation by upregulating the expression of IL-21 receptor, supporting a pivotal role of monocytes in MM disease progression (19). Macrophages from patients with MM use vasculogenic mimicry to contribute to neovessel construction following exposure to angiogenic cytokines (38).

Our knowledge regarding the heterogeneity of granulocytic and monocytic subpopulations and how innate immune population phenotypes affect the pathogenesis of MM remains limited. In this study, we investigated the heterogeneity of the phenotypic profiles of circulating neutrophilic, monocytic, and eosinophilic subpopulations in patients with MM and examined the relationship between distinct MM innate immune phenotypes and myeloma-defining events.

## Materials and methods

### Multiple myeloma study population and control selection

Eligible MM patients with histologically confirmed diagnoses enrolled in the Integrated Molecular And Genetic Epidemiology study (IMAGE) (39) were included. Patients with a diagnosis of MM were identified based on the ICD-9 classifications (203) or International Classification of Disease for Oncology third revision code 9732/3 and confirmed based on revised and updated International Multiple Myeloma Working Group classification criteria for MM (1). Myeloma was defined by the cumulative presence of clonal bone marrow plasma cells ≥ 10 percent or biopsy-proven bony or extramedullary plasmacytoma and the presence of one or more MM defining events including organ damage (hypercalcemia, renal insufficiency, anemia, or lytic bone lesions or severe osteopenia, or pathologic fractures attributed to plasma cell proliferative disorder), clonal bone marrow plasma cells ≥ 60 percent, serum involved to uninvolved FLC ratio > 100, or more than one focal bone lesion (> 5 mm) identified as previously reported (1). Each MM case was reviewed by an expert panel to ensure consistent case definitions and to minimize phenotype misclassification. Patients with extramedullary or solitary plasmacytoma or other plasma cell proliferative disorders were excluded (n=2). An additional 2 MM patients were excluded based on treatment status. After eligibility screening, a total of 35 MM patients, all previously treated as specified in **Table 1**, were included in this investigation.

**Table 1:** Control and multiple myeloma patient demographics and diagnostic clinical and laboratory characteristics of MM patients by MM1 and MM2 phenotypes.

	Combined Population					
Clinical features and laboratory characteristics (%)	Controls N=19	Total MM Cases N=35	*P*	MM1 Phenotype N=22 (62.9)	MM2 Phenotype N=13 (37.1)	* P*
Demographic characteristics						
Male sex, N (%)	10 (52.6)	17 (48.6)	0.78	9 (40.9)	8 (61.5)	0.24
Black race, N (%)	11 (57.9)	12 (34.3)	0.09	7 (31.8)	5 (38.5)	0.69
Age, median (range)	54 (43–65)	66 (43–79)	**<0.0001**	64 (43–79)	68 (58–76)	0.12
Laboratory parameters, median (range)*						
Clonal bone marrow plasma cells (BMPC), %		50 (3–97)		60 (3–97)	40 (10–80)	0.35
Calcium, mg/dL		9.6 (7.6-15.5)		9.1 (7.6-14.9)	10.4 (9.5-15.5)	0.08
Albumin, mg/dL		3.5 (1.7-4.7)		3.6 (1.7-4.5)	3.5 (2.4-4.7)	0.90
Creatinine, mg/dL		1.0 (0.5-5.2)		0.9 (0.5-5.2)	1.4 (0.7-4.2)	0.15
Hemoglobin, g/dL		10.9 (6.5-14.9)		11.1 (7.6-14.9)	10.5 (6.5-14.2)	0.30
β2-microglobulin, mg/L		3.7 (1.1-23.9)		3.6 (1.4-21.0)	5.3 (1.1-23.9)	0.48
Lactate Dehydrogenase (LDH), U/L		165 (110–355)		177 (130-355)	151 (110-332)	0.51
Monoclonal protein, total g (dL)		2.3 (0.3-5.8)		2.3 (0.3-4.0)	2.6 (0.3-5.8)	0.71
Paraprotein Assessment						
Myeloma type, N (%)						
IgG		17 (48.6)		10 (45.5)	7 (53.9)	
IgA		11 (31.4)		8 (36.4)	3 (23.1)	
Light chain restricted		7 (20.0)		4 (18.2)	3 (23.1)	0.71
FLC type, N (%)						
Kappa		20 (57.1)		10 (45.5)	10 (76.9)	
Lambda		15 (42.9)		12 (54.6)	3 (23.1)	0.07
Involved: uninvolved FLC ratio ≥ 100, N (%)		12 (41.4)		3 (16.7)	9 (81.8)	**0.001
End organ damage, N (%)						
Hypercalcemia		6 (17.1)		1 (4.6)	5 (38.5)	**0.01
Renal involvement		7 (20.0)		4 (18.2)	3 (23.1)	0.73
Anemia		19 (54.3)		12 (54.6)	7 (53.9)	0.97
Bone		20 (57.1)		12 (54.6)	8 (61.5)	0.69
International Staging System-Revised						
I		7 (21.2)		4 (19.1)	3 (25.0)	
II		18 (54.6)		13 (61.9)	5 (41.7)	
III		4 (24.2)		4 (19.1)	4 (33.3)	0.51
Treatment, N (%)						
Biologics (mAbs)		12 (34.3)		6 (27.3)	6 (46.2)	
Autologous hematopoietic stem cell transplant		13 (37.1)		7 (31.8)	6 (46.2)	
Maintenance therapy		8 (22.9)		7 (31.8)	1 (7.7)	
Pharmacologic and radiation		2 (5.7)		2 (9.1)	0 (0)	0.21

*Laboratory features determined from serum.

P-values adjusted for sex, age, race, and treatment type: Involved : uninvolved FLC ratio Padj = 0.009; hypercalcemia Padj = 0.23.

Bolded p-values are statistically significant.

Diagnostic and defining clinical features including clonal bone marrow plasma cells (%), serum monoclonal (M)-protein, involved to uninvolved FLC ratio > 100, immunoglobulin (Ig) isotype (IgG, IgA), clonality (kappa, lambda), end-organ damage [hypercalcemia (serum calcium, >11.5 mg/dl), renal insufficiency (serum creatinine, >177.0 μmol/L (>2 mg/dl) or estimated creatinine clearance <40 mL/min per 1.73 m^2^), anemia (normochromic, normocytic with hemoglobin >2 g/dl below the lower limit of normal or hemoglobin <10 g/dl)], bone involvement (radiologic evidence of lytic lesions, severe osteopenia or pathologic fractures^1^), and the revised and updated International Staging System (R-ISS) (1), and the Durie Salmon (DS) staging system (40) were determined by laboratory studies, medical history or physical examination as appropriate.

Controls were recruited through the 1917 Clinic at Dewberry at the University of Alabama at Birmingham. Eligible controls were 43 years of age and older without a history of monoclonal gammopathy of undetermined significance, smoldering multiple myeloma, MM, or other cancers. Participant characteristics are summarized in **Table 1**.

### Sample collection

All methods were performed in accordance with the relevant guidelines and regulations. Peripheral blood was collected by certified phlebotomists in tubes containing acid citrate dextrose (ThermoFisher, Waltham, MA) from MM patients or controls following informed consent. Data acquisition was performed by the IMAGE study team. Study protocols were approved by the Institutional Review Board of the University of Alabama at Birmingham (IRB protocols 141218001 and 071106009).

### Materials

A/B human serum was purchased from ThermoFisher. All solutions and materials for cell counting and the cell counter were purchased by Nexcelom (Lawrence, MA). Antibodies for flow cytometry were purchased from BioLegend (San Diego, CA) unless indicated otherwise (**Supplemental Table 1**). Dimethyl sulfoxide (DMSO), Dulbecco’s phosphate buffer solution (DPBS), and Ethylenediaminetetraacetic acid (EDTA) were purchased from Corning (Corning, NY). Phorbol myristate acetate (PMA) (Sigma Aldrich, St. Louis, MO) was dissolved in DMSO at a concentration of 1mg/ml. 2’,7’-dichlorodihydrofluorescein (H2-DCFDA) (Sigma Aldrich) was dissolved in DMSO at a concentration of 1mM. All antibody stain panels were made in 10% A/B human serum in DPBS.

### Whole blood staining

Twenty milliliters of ACD-treated blood was collected from MM patients or controls and processed within three hours of the collection as described previously (41). Briefly, fifty microliters of whole blood were stained for 30 minutes with 50 μl of pre-mixed antibodies for the base panel and whole blood staining (**Supplementary Table 1**) at 4°C. Samples were washed with 4 ml 0.1M EDTA in DPBS and centrifuged at 200 x g for 5 minutes. Red blood cell lysis and fixation was performed in 1 ml of 1x 1-step Fix/lyse buffer (Invitrogen, Waltham, MA) at room temperature for 15 minutes. Samples were washed with 2% fetal bovine serum (FBS) (Atlanta Biologicals, Atlanta GA) in DPBS, centrifuged for 5 minutes at 200 x g, and suspended in equal parts 2% FBS in DPBS and intracellular fixation buffer (IC fix) (Invitrogen). All samples were held at 4°C prior to analysis, filtered with 40 μm mesh, and acquired on the Attune NxT flow cytometer (ThermoFisher) within 24 hours of processing, and FlowJo V 10.7 (FlowJo LLC, Ashland, OR) was used for data analysis. Determination of the levels of surface antigens using acoustic self-focusing technology may result in negative values following background subtraction; however, the resulting values retain biological significance. Fluorescent minus one (FMO) controls were employed to account for autofluorescence and nonspecific signals. The percentage of CD64^neg^ cells was determined as events below the gate set at 95% of events of FMO control from a healthy donor (**Supplemental Figure 2B**).

### Processing and staining of peripheral blood mononuclear cells

Four ml of whole blood was diluted with 4 ml of DPBS and layered onto 4 ml of discontinuous Ficoll-Paque PREMIUM density gradient (1.078g/ml) (GE Healthcare, Chicago, IL). The samples were centrifuged for 30 minutes at 400 x g. The PBMC layer was isolated into 10 ml of DPBS, centrifuged at 300 x g for 10 minutes, washed with 10 ml of DPBS, and centrifuged at 200 x g for 10 minutes. PBMCs were suspended in 10% A/B human serum in DPBS. Twenty μl of cell suspension was stained with 20 μl Viastain™ Acridine Orange/Propidium Iodide Staining Solution for 2 minutes at RT. 20 μl of stained cells were loaded on a hemocytometer and counted using the Cellometer K2 Fluorescent Viability Cell Counter. Aliquots of 1 x 10^6^ PBMCs/50 μl were suspended in 10% A/B human serum and incubated at 4°C for 30 minutes. Samples were stained with 50 μl of pre-mixed antibodies for the base panel and PBMC staining (**Supplementary Table 1**) at 4°C for 30 minutes, washed with 2% FBS in DPBS, and suspended in equal parts 2% FBS in DPBS and IC fix. Sample acquisition and analysis of LDNs within the PBMC sample were performed as described in “whole blood staining.”

### Gating strategy for phenotype characterization

Single cells were identified and doublets were removed using forward scatter height and area. For all phenotypic analyses, CD3^+^ and CD19^+^ events corresponding to T and B lymphocytes, respectively, were removed. For monocyte analyses, CD14 and CD16 were utilized following the removal of CD15^+^ and CCR3^+^ events. Patrolling monocytes (Pt Mo) were identified as CD16^high^CD14^low^ and classical monocytes (Cl Mo) as CD16^low^CD14^high^ (**Supplemental Figure 1**). Since the intermediate monocyte population defined as CD16^high^CD14^high^ was not clearly defined in most patients and was highly affected by shifts in the expression of CD16 on classical monocytes, a separate examination of the intermediate monocyte population was not included in the final analysis. For whole blood neutrophil and LDN analyses, CD193^+^ (CCR3) and CD14^+^ events were removed. Mature neutrophils (mNs) were identified as CD16^high^ and immature neutrophils (imNs) as CD16^-^. For eosinophils, CCR3^+^ and CD15^+^ were analyzed following the removal of CD14^+^ and CD16^+^ events (**Supplemental Figure 1**).

### Quantification of innate immune cells

Fifty microliters of fresh whole blood from healthy or MM patients were stained with 50 μl of a separate pre-mixed antibody absolute count panel for 30 minutes at 4°C (**Supplemental Table 1**). Red blood cell lysis and fixation were performed in 1 ml of 1x 1-step Fix/lyse buffer at RT for 15 minutes. 50 μl of CountBright™ Absolute Counting Beads (ThermoFisher) were added to samples using a pre-determined concentration given by the manufacturer. Samples were held at 4°C prior to acquisition and acquired on the Attune NxT flow cytometer (ThermoFisher) within 24 hours of processing and analyzed with FlowJo V 10.7 (FlowJo LLC). Bead counts were determined per the instructions by the manufacturer. Briefly, the bead adjustment factor was calculated by dividing the number of beads gated by the pre-determined concentration of beads per 50 μl. Leukocytes were gated with side scatter and forward scatter area and total neutrophils were gated using CD15^+^CD14^-^ gate with adjustment performed by multiplying cell counts by the bead adjustment factor.

For quantification of neutrophil or monocyte subsets in whole blood, the percentages of the subset gate from total neutrophils (imN and mN) or monocytes (Pt Mo and Cl Mo) were multiplied by the adjusted total neutrophil or monocyte count. A count threshold of 150 imN per sample was determined based on the calculated mean imN frequency of samples. The majority of healthy controls did not meet the event threshold for imNs and were excluded in the phenotypic analyses.

For the quantification of neutrophils in the PBMC layer, the percentage of total LDNs was gated and multiplied by the percentage of total neutrophils in whole blood. LDN subsets were quantified by multiplying the percent of the subset of total neutrophils by the PBMC neutrophil count.

### Adjustment of median fluorescent intensities for phenotypic analyses

The SPHERO™ Ultra Rainbow Calibration Particle Kit (Spherotech Inc., Lake Forest, IL) was utilized to assess variation in laser fluorophore intensities with rainbow beads acquired at each time of sample acquisition. Histogram overlays including rainbow beads acquired at baseline and each subsequent sample after were generated of each fluorophore. The need for MFI adjustment was determined by the difference of emission peaks varying by a half log or greater. If adjustments were needed, histogram emission peaks 1-6 were gated for each fluorophore, as demonstrated in the company protocol, and the mean MFI was determined for each peak. The difference between peak MFIs was calculated by subtracting the current MFI from the baseline MFI. The percentage difference was determined by dividing the mean MFI difference by the MFI at baseline for each peak. Mean peak percent difference was determined by averaging percentages of peaks 3-6 for each fluorophore. MFIs of specific markers were then multiplied by the percentage corresponding to the fluorophore the marker was conjugated to, resulting in the normalized MFI.

### Isotonic lysis of cells

Two milliliters of isotonic lysis buffer (155 mM NH_4_Cl, 10mM KHCO_3_, 0.1mM EDTA in water sterile filtered with a Steritop 0.22µm filter) (26) was added to 50-100 μl of whole blood and set on a rotator for 5 minutes or until the red blood cells were lysed. Two milliliters of 2% FBS in DPBS was added and samples were centrifuged for 5 minutes at 200 x g. The pellet was suspended in 500 μl of 2% FBS in DPBS for immediate acquisition for functional assays or 10% human A/B serum in DPBS for further processing for proliferation assessment.

### Assessment of intracellular expression of Ki67

Intracellular staining was performed using the Cytofix/Cytoperm™ Fixation/Permeabilization Kit (BD Biosciences). One hundred microliters of whole blood from MM patients were isotonically lysed as described in “isotonic lysis of cells.” PBMCs were isolated, and 2.5 x 10^5^ PBMC aliquots were suspended in 50 μl of 10% A/B human serum in DPBS for 30 minutes at 4°C. Fifty microliters of the extracellular Ki67 antibody panel were added for 30 minutes at 4°C (**Supplementary Table 1**). The fixation and permeabilization were performed according to the manufacturer’s protocol. Samples were then stained with 5 μl of Ki67 antibody for 30 minutes at 4°C. Samples were washed with 2% FBS in DPBS and centrifuged for 5 minutes at 200 x g. This step was repeated. The final pellet was suspended in equal parts 2% FBS in DPBS and IC fix. Sample acquisition and analysis were performed as described in above with a final cytogram visualized by side scatter and Ki67 and quartile gate applied using a fluorescence minus one (FMO) for APC to set the negative gate and determine Ki67-positive cells.

### LEGENDplex™ protocol for measuring plasma markers and cytokine levels in plasma

The LEGENDplex™ Human Essential Immune Response Panel 740929 13-plex kit and LEGENDplex™ customization kit, including CCL2, CCL11, CRP, CXCL10, D-dimer, G-CSF, IFNγ, IL-18, IL-1β, IL-6, CXCL9, and TNF-R1 were utilized (BioLegend). Plasma samples were stored at -80°C. For the assay, the samples were thawed, centrifuged for 10 minutes x 500 g, and filtered using the filter plate for LEGENDplex™ Assay (BD Biosciences). Samples were processed according to the manufacturer’s instructions for the filter plate assay. In brief, 25 μl aliquots were added to the 96-well plate with 25 μl assay buffer for samples, 25 μl of Matrix B for standards, and 12.5 μl of the pre-mixed immune bead solution at RT covered on a shaker for 2 hours in the dark. Unbound beads were removed with gentle vacuum pressure and then washed with 1X wash buffer. 12.5 μl of detection antibody was added to samples and incubated for 1 hour covered on a shaker at RT in the dark. 12.5 μl of SA-PE was added to samples and incubated for 1 hour covered and shaken at RT in the dark. Samples were washed with 1X wash buffer and manually transferred to FACs tubes with a final volume of 150 μl per sample. The template for data acquisition and instrument setup was performed as described in the manufacturer’s protocol on the BD Symphony™ (BD Biosciences) the same day. Analyses were performed utilizing the online LEGENDplex™ Data Analysis Software (BioLegend).

### Determination of the levels of soluble plasma markers by ELISA

Plasma was collected after single discontinuous density gradient centrifugation as described in “the processing of peripheral blood mononuclear cells (PBMCs)” and stored at -80°C until use. Plasma levels of sCD14 and lipocalin-2 (NGAL) were measured using commercial ELISA kits Hycult HK320 and Hycult HK330 (Hycult Biotechnology, Uden, the Netherlands), and sCD163 using commercial ELISA kit Quantikine DC1630 (R&D Systems, Minneapolis, MN) according to the manufacturer’s instructions. The final dilution ratio was 1:80 for sCD14 and NGAL and 1:10 for sCD163. Absorbance was measured with the ELx808™ Biotek absorbance microplate reader (Biotek, Winooski, WT), and concentrations were calculated using Gen5™ data analysis software (BioTek) based on a standard curve.

### Assessment of ROS and mitochondrial superoxide production

Fifty microliters of whole blood or isolated PBMCs (1 x 10^6^ aliquots per 50 μl) were stained with the ROS antibody panel in pre-warmed tubes (**Supplemental Table 1**). A final concentration of 20µM of H2-DCFDA was added, and samples were simultaneously stimulated with or without 10nM of PMA. Samples were incubated at 37°C for 30 minutes. Samples were lysed as described in “isotonic lysis of cells,” acquired within 30 minutes after preparation, and analyzed *via* flow cytometry. The same protocol was performed for superoxide production, utilizing the MitoSOX antibody panel, and a final concentration of 2mM of MitoSOX™ Red (Invitrogen) was added to samples simultaneously with PMA stimulation (**Supplemental Table 1**).

### Assessment of mitochondrial mass and mitochondrial activity

Fifty microliters of fresh whole blood or isolated PBMCs (1 x 10^6^ cells per 50 μl sample) from MM patients or controls were stained with the MitoTracker Green antibody panel using pre-warmed tubes (**Supplemental Table 1**). A final concentration of 1 μM of MitoTracker™ Green FM (MTG) (Invitrogen) was added to tubes to determine mitochondrial mass. Simultaneously, a final concentration of 500nM tetramethylrhodamine ethyl ester (MitoStatus TMRE) (BD Biosciences) was added to determine mitochondrial membrane potential (ΔΨm). Samples were incubated at 37°C for 30 minutes and lysed as described in “isotonic lysis of cells.” The samples were analyzed by flow cytometry within 30 minutes. ΔΨm was adjusted to the mitochondrial mass of a corresponding sample and presented as ΔΨm/MTG ratio.

### Assessment of phagocytosis

Fifty microliters of fresh whole blood or isolated PBMCs (1 x 10^6^ per 50 μl sample) from MM patients or HD was stained with a premade antibody mix base panel and the pHrodo antibody panel using pre-warmed tubes (**Supplemental Table 1**). 10 μl of pHrodo™ Red *E. coli* BioParticles™ (ThermoFisher) was added to determine the phagocytic capacity of leukocytes as indicated by an increase in pHrodo™ red fluorescence indicating a decrease in pH. Samples were incubated at 37°C for 30 minutes. Samples were processed as described in “whole blood staining.”

### Statistical analysis

Tests for statistical significance of myeloid phenotype marker expressions (*p*<0.05) were conducted using the Mann-Whitney rank-sum test between MM cases and controls. Correlations between marker expression and clinical parameters were evaluated using the Spearman rank-order test. Tests for statistical significance of marker expressions stratified by disease stage were performed using the Kruskal-Wallis one-way ANOVA followed by Dunn’s Multiple Comparison *post hoc* test. The myeloid phenotype of MM patients relative to clinical parameters was calculated using logistic regression adjusted for confounders (race, sex, age, and treatment type). All calculations were performed with GraphPad Prism (GraphPad Software Inc., La Jolla, CA) or Stata v.16.0 (StataCorp, College Station, TX).

## Results

### A subset of MM patients demonstrates an altered neutrophil phenotype

**Figure 1:**
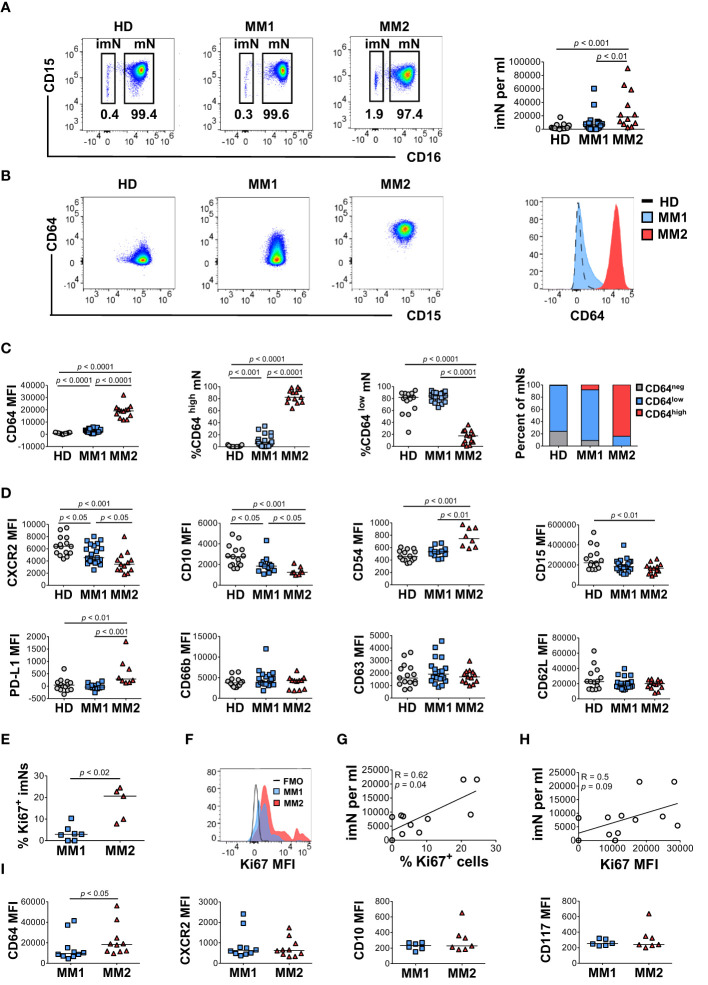
A subset of MM patients exhibits an altered neutrophil phenotype. **(A)** Left panel: representative cytograms of CD15^+^CD16^+^ mature neutrophils (mNs) and CD15^+^CD16^-^ immature neutrophils (imNs) in whole blood of a healthy donor (HD), MM1 patient, and MM2 patient. Right panel: frequency of imN per ml of blood of MM1 and MM2 patients and HD. **(B)** Representative cytograms of mNs of HD and MM1 and MM2 patients and a histogram overlay of CD64 expression. **(C)** Levels of expression of CD64 and percentages of CD64^high^ and CD64^low^ mNs in HD, MM1 and MM2 patients. **(D)** Levels of expression of maturation and activation markers on mNs of HD, MM1 and MM2 patients. **(E)** Percentage of Ki67^+^ imNs of total imNs. **(F)** Histogram overlay of Ki67 intracellular expression on imNs and **(G)** a correlation between imN frequency and the percentage of Ki67^+^ imNs, n=11. **(H)** Correlation between imN frequency and MFI of Ki67^+^ imNs, n=11. **(I)** Levels of expression of maturation and activation surface markers on imNs of MM1 and MM2 patients. MFI, median fluorescent intensity. Statistical analyses were performed using the Mann-Whitney rank-sum test **(A, C–E** and **I)** or Spearman correlation **(G, H)**. Spearman correlation coefficients R and p values are indicated; bars and lines represent median values and simple linear regression analysis, respectively.

To determine the heterogeneity of the myeloid cell compartment in the peripheral blood of patients with MM, whole blood cells were characterized by multiparametric flow cytometry using antibodies designed to assess myeloid cell differentiation and activation (**Supplemental Table 1**; **Supplemental Figures 1** and **2**). Neutrophil phenotype was assessed on two distinct neutrophil subsets, CD16^neg^ immature neutrophils (imN) and CD16^pos^ mature neutrophils (mN) (**Supplemental Figure 1**; **Figure 1A**). Comprehensive analyses revealed two distinct phenotypes characterized by specific patterns of surface antigen expression on neutrophils, monocytes, and eosinophils. A subset of MM patients exhibited significantly higher surface levels of CD64 (FcγRI) on CD16^pos^ circulating mNs (**Figures 1A–C**) (42, 43). MM patients exhibiting CD64 expression on mNs equal to or higher than 15 standard deviations from the mean value detected in HDs were included in the MM2 (CD64^high^) group; patients with mN CD64 expression below this level were included in the MM1 (CD64^low^) group (**Figures 1B, C**). MM2 (CD64^high^) phenotype was observed in 13 out of 35 patients (37%). The percentage of CD64^neg^ cells was highest in HDs and lowest in MM2 patients (**Supplemental Figure 2B**). The lower maturation status of mNs from MM2 (CD64^high^) patients is indicated by increased CD64 levels and reduced levels of CXC chemokine receptor 2 (CXCR2), CD10, and CD15 compared to neutrophils from MM1 patients or healthy donors (**Figure 1D**). Neutrophils from MM2 patients displayed elevated levels of PD-L1 relative to MM1 (*p*<0.001) or controls (*p*<0.01) suggesting enhanced immunosuppressive potential (16, 17, 23, 44) (**Figure 1D**). There was no indication of neutrophil activation as determined by an absence of changes in the surface levels of CD16 or lectin-type oxidized LDL receptor 1 (Lox-1) and an absence of surface shedding of CD31 or CD62L (42, 45–47) (**Figure 1D** and **Supplemental Figure 3A**). Similarly, no indication of neutrophil degranulation was observed on neutrophils from MM2 patients as determined by an absence of changes in the levels of degranulation markers myeloperoxidase (MPO), CD11b, CD63, or CD66b (48–51) (**Figure 1D** and **Supplemental Figure 3A**). The neutrophil: lymphocyte ratio did not significantly differ between controls, MM1, and MM2 patients (**Supplemental Figure 3B**). The presented data indicate the presence of a phenotypically distinct and alternatively differentiated mature neutrophil population in MM2 patients that does not exhibit typical signs of activation or degranulation.

### CD16^neg^ immature neutrophils are expanded in the blood of MM2 patients

Immature neutrophils (imNs) consist of circulating neutrophil progenitor populations characterized by the SSC^high^CCR3^-^CD10^low^CD15^+^CD16^neg^ phenotype (42, 52, 53). imNs are expanded in patients with a variety of chronic inflammatory diseases and multiple types of cancer (15, 42, 54–57). MM2 patients exhibit a significant expansion of CD16^neg^ imNs relative to MM1 patients (*p*<0.01) or controls (*p*<0.001; **Figure 1B**, right panel). imNs from MM2 patients display higher intracellular expression of a proliferation marker Ki67 relative to MM1 (*p*<0.02; **Figures 1E, F**). A significant positive correlation was observed between the frequency of imNs and the percentage of Ki67^+^ imNs (**Figure 1G**; correlation coefficient R=0.62, *p*=0.04), and a trend for a positive correlation was observed between imN frequency and Ki67 expression (**Figure 1H**). The percentage of Ki67^+^ mNs did not significantly differ between MM1 and MM2 patients (**Supplemental Figure 3D**). imNs expressed higher levels of CD64 on MM2 relative to MM1 (*p*<0.05) with no significant difference in the expression of other markers tested (**Figure 1I** and **Supplemental Figure 3E**).

### Mature low-density neutrophils are expanded and exhibit a distinct phenotype in MM2 patients

**Figure 2:**
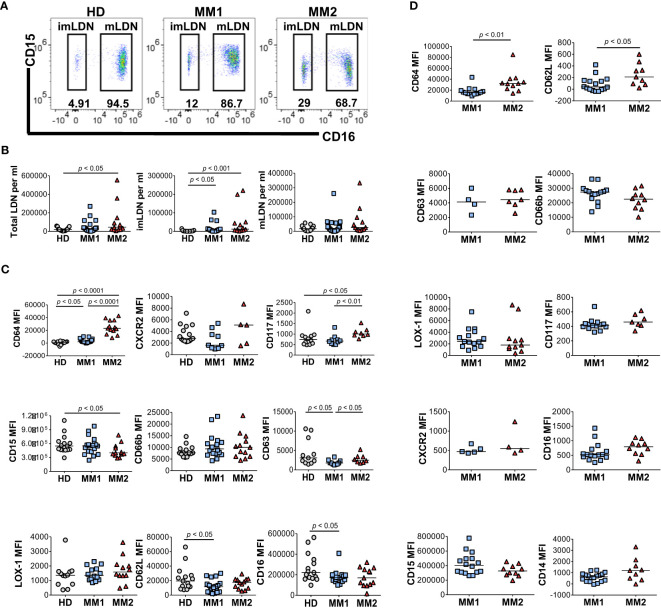
Distinct phenotypes on low-density neutrophils obtained from MM1 and MM2 patients. **(A)** Representative cytograms of CD15^+^CD16^+^ mature low-density neutrophils (mLDNs) and CD15^+^CD16^-^ immature low-density neutrophils (imLDNs). **(B)** Frequencies of total LDNs, mLDNs, and imLDNs in the blood of HD, MM1, and MM2 patients. **(C, D)** Surface expression of maturation, degranulation, and activation markers on mLDNs **(C)** and imLDNs **(D)** from MM1, and MM2 patients. MFI, median fluorescent intensity; statistical analyses were performed using the Mann-Whitney rank-sum test with *p* values indicated. Bars represent median values.

Low-density neutrophils (LDNs) or PMN-MDSCs co-localizing with lymphocytes following cell separation by density gradient centrifugation are expanded in chronic inflammatory conditions and cancer and exert significant immunosuppressive properties (15, 23, 28, 44, 52, 58). Significantly higher frequencies of total LDNs (*p*<0.05) and CD16^neg^ immature LDNs (imLDNs) (*p*<0.001) were observed in MM2 patients compared to controls (**Figures 2A, B**). Mature LDNs (mLDNs) from MM2 patients exhibited less differentiated neutrophil phenotype relative to MM1 patients as indicated by elevated CD117 (*p*<0.01) and CD64 (*p*<0.0001) (**Figure 2C**) (54, 55, 59). Consistent with the findings in whole blood, mLDNs from MM2 patients exhibited a significant decrease in CD15 expression relative to controls (*p*<0.05; **Figure 2C**). imLDNs from MM2 exhibited significantly higher expressions of CD64 (*p*<0.01) and CD62L (*p*<0.05) relative to MM1 (**Figure 2D**).

### MM2 patients demonstrate altered monocytic and eosinophilic phenotypes

**Figure 3:**
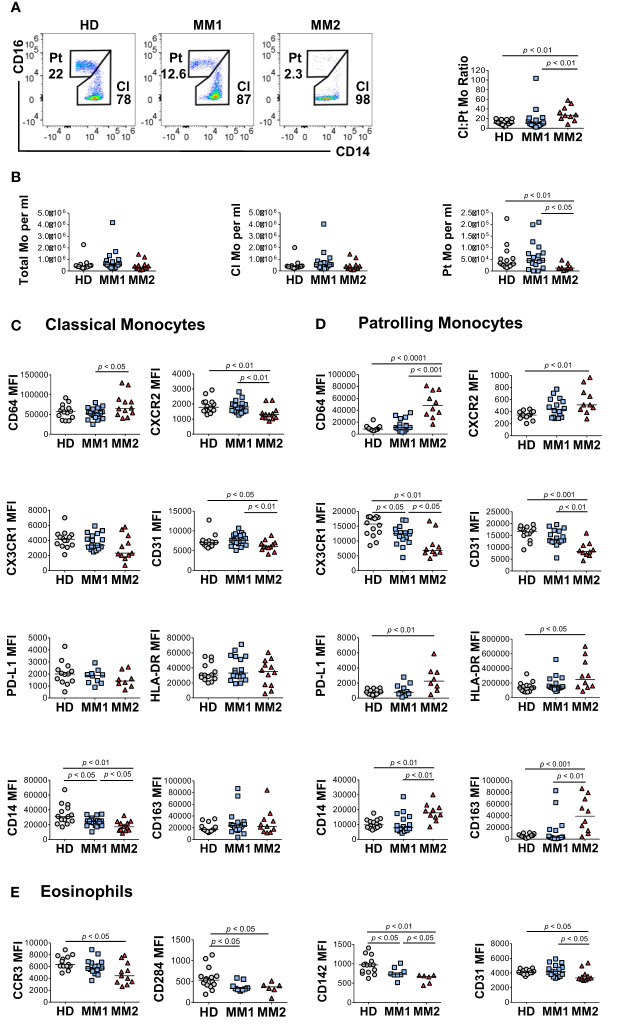
Altered monocytic and eosinophilic phenotypes in MM2 patients. **(A)** Left panel: representative cytograms of CD16^low^CD14^high^ classical monocytes (Cl Mo) and CD16^high^CD14^low^ patrolling monocytes (Pt Mo) in whole blood from HD, MM1 and MM2 patients. Right panel: classical to patrolling monocyte ratios from HD, (*n*=15) MM1, (*n*=16) and MM2 (*n*=10) patients. **(B)** Frequencies of total, classical, and patrolling monocytes. **(C, D)** Surface levels of monocyte markers on classical **(C)** or patrolling monocytes **(D)**. **(E)** Surface levels of eosinophil markers of HD, MM1, and MM2 patients. MFI, median fluorescent intensity; statistical analyses were performed using the Mann-Whitney rank-sum test with *p* values indicated. Bars represent median values.

To address monocytic heterogeneity in MM, the phenotypes of classical (CD16^low^CD14^high^) and patrolling (CD14^low^CD16^high^) monocyte subpopulations were determined (60, 61). MM2 patients exhibited a significantly lower frequency of patrolling monocytes relative to MM1 (*p*<0.05) or controls (*p*<0.01; **Figures 3A, B**). There were no significant differences in the frequency of total monocyte population, frequency of M-MDSCs, or lymphocyte: monocyte ratios between MM2 patients and HD (**Figure 3B** and **Supplemental Figure 3C**). The intermediate monocyte population defined as CD16^high^CD14^high^ was not clearly defined in most patients and was highly affected by the shifts in the expression of CD16 on classical monocytes; it was therefore not included in the final analysis. Consistent with the mature neutrophil phenotype, classical monocytes from MM2 patients exhibited significantly higher expression of CD64 (*p*<0.05) and lower levels of CXCR2 (*p*<0.01) compared to MM1 (**Figure 3C**). Patrolling monocytes from MM2 patients exhibited higher levels of CD64 relative to MM1 patients (*p*<0.001) and controls (*p*<0.0001; **Figure 3D**). In contrast, CXCR2 levels on patrolling monocytes from MM2 patients were significantly higher relative to controls (*p*<0.01; **Figure 3D**).

An activated monocytic phenotype was observed in MM2 relative to MM1 patients indicated by a significant decrease in CD31 expression on classical and patrolling monocytes (**Figures 3C, D** and **Supplemental Figure 4**). Additionally, patrolling monocytes from MM2 patients demonstrate a significant increase in activation markers CD163 (*p*<0.01) and CD169 (*p*<0.01; **Figure 3D** and **Supplemental Figure 4F**). Patrolling monocytes from MM2 exhibited downregulation of CX3 chemokine receptor 1 (CX3CR1) (*p*<0.01) and upregulation of PD-L1 (*p*<0.01) relative to controls consistent with the features observed in an immunosuppressive environment (23, 26) (**Figure 3D**).

Eosinophils were shown to accelerate MM progression in synergy with microbiota-driven IL-17-producing cells in murine models (62). Decreased C-C Chemokine receptor 3 (CCR3) expression on eosinophils was observed in MM2 patients compared to controls (*p*<0.05; **Figure 3E**). Eosinophils from MM2 patients exhibited lower levels of cell signaling receptor tissue factor (CD142) (63) (*p*<0.05), activation marker CD66b (64) (*p*<0.05) and CD31 (*p*<0.05) relative to MM1 patients (**Figure 3E** and **Supplemental Figure 5**).

### Myeloid cells in MM2 patients demonstrate altered functional activity

**Figure 4:**
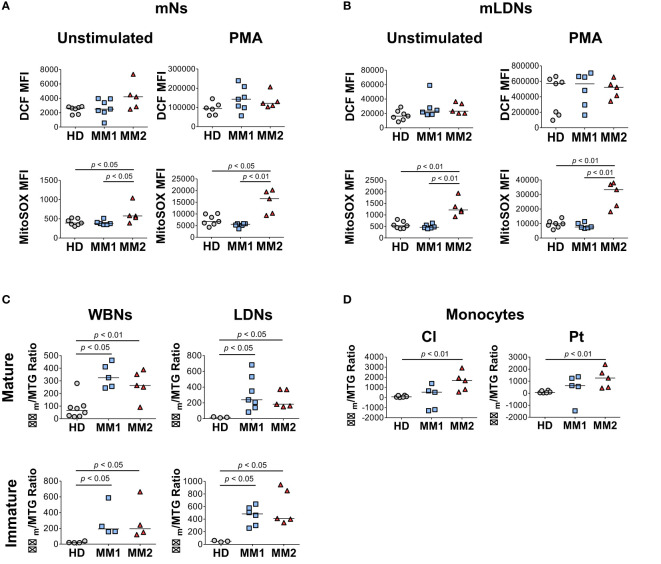
Myeloid cells obtained from MM2 patients exert altered functional activity. **(A, B)** Reactive oxygen species (ROS) production determined by dichlorodihydrofluorescein (DCF) staining and mitochondrial superoxide production by MitoSOX™ Red at baseline and upon stimulation with phorbol myristate acetate (PMA) in mNs **(A)** and mLDNs **(B)** of HD (*n*=8) and MM1, (*n*=5) and MM2 (*n*=5) patients. **(C, D)** Mitochondrial membrane potential was determined using tetramethylrhodamine ethyl ester (TMRE) staining adjusted for mitochondrial mass as detected by MitoTracker™ Green (Δψm/MTG) on neutrophils **(C)** and monocytes **(D)**. MFI, median fluorescent intensity; statistical analyses were performed using the Mann-Whitney rank-sum test with *p* values indicated. Bars represent median values.

Next, the differences in the bioenergetic properties of myeloid cells in MM patients were assessed. Neutrophils from MM2 patients exhibited significantly higher mitochondrial superoxide production detected using MitoSOX™ Red fluorogenic dye in the absence and presence of PMA stimulation compared to neutrophils from MM1 patients or controls (**Figure 4A**). Similar results were observed in the LDN subpopulations suggesting that neutrophils from MM2 patients exert higher capacity for mitochondrial superoxide release (**Figure 4B**). Neutrophil subpopulations from both MM1 and MM2 patients demonstrated significantly higher mitochondrial potential detected using the cationic TMRE dye sequestered by mitochondria with adjustment for mitochondrial mass indicated by MitoTracker™ Green staining relative to controls (*p*<0.05; **Figure 4C**). Classical and patrolling monocytes from MM2 patients exhibited increased mitochondrial potential relative to controls (*p*<0.01; **Figure 4D**). mLDNs from MM2 patients demonstrated higher phagocytic capacity as determined by the phagocytosis of pHrodo™ Red E. coli-conjugated BioParticles™ relative to controls (*p*<0.05; **Supplemental Figure 6A**). No differences were observed in the phagocytic capacity of monocyte subpopulations between MM1 and MM2 patients (**Supplemental Figure 6B**).

### Soluble markers of myeloid cell activation are significantly elevated in MM2 patients

**Figure 5:**
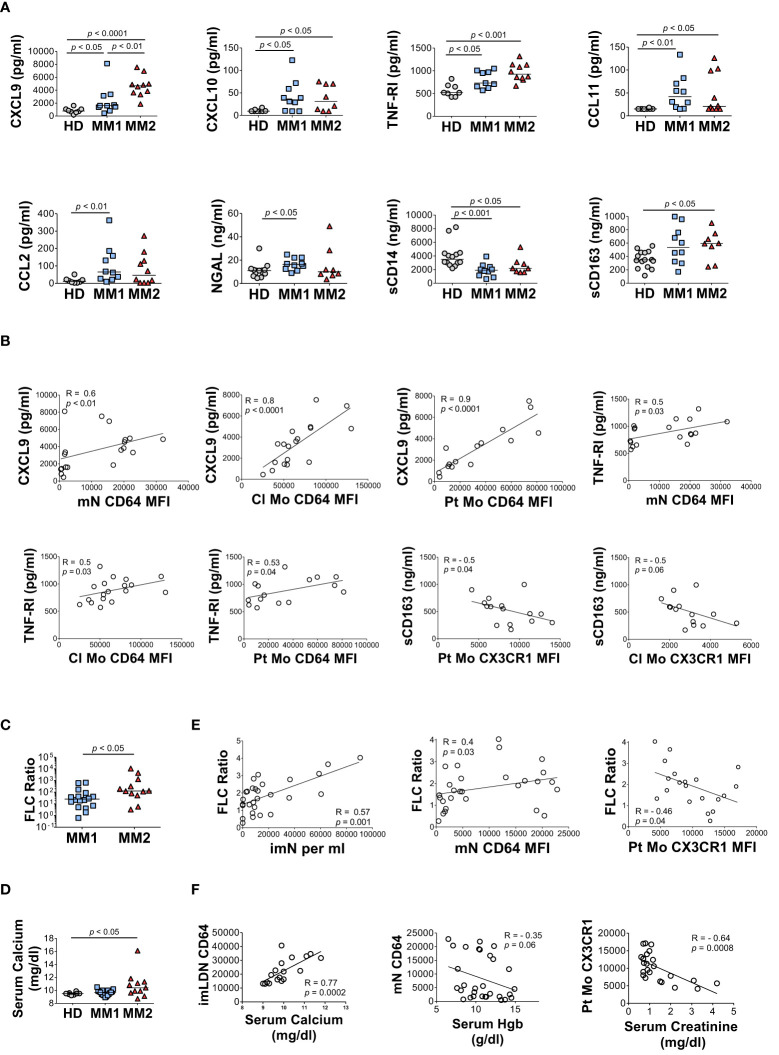
The MM2 phenotype is associated with changes in the plasma levels of markers of inflammation and with clinical markers of disease progression. **(A)** Levels of cytokines and markers of myeloid activation in plasma of HD and MM1 and MM2 patients. **(B)** Correlations between the plasma levels of markers of myeloid activation and surface levels of antigens on mN and patrolling monocytes. **(C)** (involved/uninvolved immunoglobulin free light chain) FLC Ratio of MM1 and MM2 patients. **(D)** Serum calcium levels of HD, MM1, and MM2 patients. **(E)** Correlation between the FLC ratio and characteristic features of the MM2 phenotype (*n*=29). **(F)** Correlations between the characteristic features of the MM2 phenotype and myeloma-defining events (*n*=24). Serum calcium levels adjusted for albumin [(0.8 x (4g/dl*normal albumin – patient albumin)) + patient serum calcium level]. MFI, median fluorescent intensity. **(A, C, D)** or the Spearman correlation test **(B, E, F)**. Spearman correlation coefficients R and *p* values are indicated; bars and lines represent median values and simple linear regression analysis, respectively.

MM2 patients exhibited higher plasma levels of several key markers of myeloid cell activation. CXCL9 (monokine induced by interferon-gamma; MIG) levels were significantly higher in MM2 relative to MM1 patients (*p*<0.01) or controls (*p*<0.0001; **Figure 5A**). Both MM1 and MM2 patients displayed higher levels of CXCL10 (IP-10) and tumor necrosis factor receptor 1 (TNF-R1) relative to controls (**Figure 5A**). The levels of CXCL9 and TNF-R1 positively correlated with the levels of CD64 on mature neutrophils and classical and patrolling monocytes (**Figure 5B**). MM1 patients demonstrated higher levels of CCL2 (monocyte chemoattractant protein-1; MCP1), eosinophil chemotactic CC-chemokine CCL11 (eotaxin-1), and neutrophil gelatinase-associated lipocalin (NGAL) relative to controls (**Figure 5A**). There was no significant difference in plasma levels of granulocyte colony-stimulating factor (G-CSF), CRP, IL-6, IL-1β, IFNγ, and TNFα between MM1 and MM2 patients (**Supplemental Figure 7** and data not shown). To assess the level of monocyte/macrophage activation, plasma levels of soluble markers sCD14 and sCD163 were determined (65–68). The levels of sCD14 were lower in both MM1 and MM2 patients relative to controls (**Figure 5A**), corresponding to lower levels of cell-associated CD14 on classical but not patrolling monocytes (**Figures 3C, D**). In contrast, sCD163 was significantly elevated in the plasma of MM2 patients relative to controls (*p*<0.05; **Figure 5A**), consistent with a prior study (66). The level of sCD163 corresponded to elevated CD163 expression on patrolling monocytes (**Figure 3D**) and negatively correlated with the expression of CX3CR1 on patrolling and classical monocytes (**Figure 5B**).

### MM2 phenotype components and presence of clinical features

Clinical features indicative of end-organ damage at diagnosis are summarized in **Table 1**. Compared to the MM1 phenotype, significantly more patients with the MM2 (CD64 ^high^) phenotype displayed involved to uninvolved FLC ratios > 100 at diagnosis after adjusting for sex, race, age, and treatment (*p*=0.009), suggesting that the MM2 phenotype may be reflective of myeloid dysregulation in MM patients with elevated FLC ratios (**Figure 5C**; **Table 1**). MM2 patients exhibited a trend toward increased serum calcium levels relative to controls (**Figure 5D** and **Table 1**).

Myeloma-defining events were analyzed in relation to the defining characteristics of the MM2 phenotype. The involved to uninvolved FLC ratio positively correlated with the frequency of imNs (R=0.57, *p*=0.001) and CD64 expression on mNs (R=0.4, *p*=0.03) and negatively correlated with CX3CR1 expression on patrolling monocytes (R= -0.46, *p*=0.04, **Figure 5E**). High CD64 expression on imLDNs positively correlated with elevated serum calcium related to hypercalcemia (R=0.77, *p*=0.0002; **Figure 5F**). Additionally, high CD64 expression on mNs partially correlated with lower hemoglobin values used to define anemia (R= -0.35, *p*=0.06; **Figure 5G**). Levels of CX3CR1 on patrolling monocytes negatively correlated with serum creatinine levels indicating renal involvement (R= -0.64, *p*=0.0008; **Figure 5F**).

G-CSF, which is commonly administered after aHSCT, has been previously reported to induce high neutrophil CD64 expression (69–71). Although CD64 expression on mNs and classical monocytes positively correlated with the start of induction therapy, aHSCT did not notably contribute to the MM2 phenotype (**Supplemental Figures 8** and **9**). Of the twelve patients treated with monoclonal antibody-based therapy in combination with chemotherapy, six demonstrated the MM2 phenotype, with the difference between groups not reaching a level of statistical significance (**Table 1**). Logistic regression analysis demonstrated an absence of any significant association between the MM phenotype and the remission state. A significant increase in CD15 expression on mNs of MM patients with dominant lambda clonal disease was observed relative to those with kappa disease (*p*<0.001) and an increase in CX3CR1 was observed on classical monocytes obtained from patients with the IgG isotype (*p*<0.05). No other notable differences were observed in relation to the myeloma isotypes (**Supplemental Figure 10**). Lower levels of CD284 (*p*<0.05) and CD142 (*p*<0.05) were observed on eosinophils obtained from patients with a diagnosed bone disease (**Supplemental Figure 11**). No other significant differences with regard to MM2 phenotype components were observed (**Supplemental Figure 11**).

### Several defining characteristics of the MM2 (CD64 ^high^) myeloid cell phenotype are associated with an advanced disease stage

**Figure 6:**
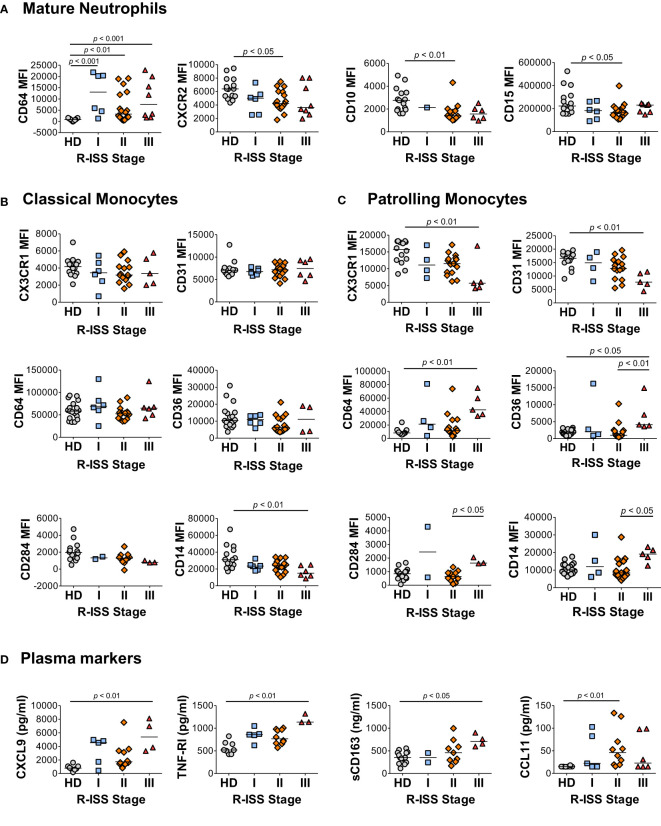
Characteristic features of the MM2 phenotype are associated with an advanced R-ISS stage. **(A–C)** Expression of surface markers on mature neutrophils **(A)**, classical monocytes **(B)**, and patrolling monocytes **(C)** stratified using R-ISS. **(D)** Levels of cytokines and plasma markers of activation stratified using R-ISS. MFI, median fluorescent intensity; statistical analyses were performed using the Kruskal-Wallis one-way ANOVA test followed by Dunn’s *post hoc* test with *p* values as indicated. Bars represent median values.

To address the relationship between the characteristic components of the MM2 (CD64 ^high^) phenotype and disease stage, granulocytic and monocytic markers of MM patients and plasma markers of myeloid activation were stratified by the revised and updated International Staging System (R-ISS) criteria with R-ISS stage III indicative of advanced disease (**Figure 6** and **Supplemental Figure 12**). Mature neutrophils from some, but not all, MM stage III patients exhibited significantly higher CD64 expression (*p*<0.001) relative to controls. Mature neutrophils from stage II patients exhibited significantly lower levels of CXCR2 (*p*<0.05) and CD10 (*p*<0.01) relative to controls (**Figure 6A**). No difference was observed in neutrophil activation and degranulation markers among MM patients (**Figure 6A**) indicating that the observed differences were not induced by neutrophil activation or degranulation. A significant decrease of CD14 expression was observed on classical monocytes (*p*<0.01) from stage III patients, while patrolling monocytes from stage III patients exhibited significantly lower levels of CX3CR1 (*p*<0.01) and CD31 (*p*<0.01) relative to controls (**Figures 6B, C**). Further, a significant increase was observed in CD64 (*p*<0.01) and CD169 (*p*<0.05) on patrolling monocytes from stage III patients relative to controls (**Figure 6C** and **Supplemental Figure 12C**). The levels of CXCL9, TNF-RI, and sCD163 were significantly elevated in stage III patients relative to HD (**Figure 6D**). Similar trends in myeloid marker expression and cytokine levels were observed utilizing the Durie-Salmon (DS) staging system, in particular an increase of CD64 and a decrease of CXCR2 and CD10 levels on neutrophils from patients with an advanced disease stage (**Supplemental Figure 13**). Overall, these data suggest that several components of the MM2 myeloid phenotype are associated with an advanced MM disease stage.

## Discussion

In this study, we report that a subset of MM patients exhibits an altered myeloid cell phenotype characterized by significant changes in the properties of circulating granulocytic, monocytic, and eosinophilic populations. Characteristic features of the observed MM2 (CD64^high^) phenotype are associated with advanced-stage disease and myeloma-defining events, including hypercalcemia and elevated involved-to-uninvolved free light chain ratio. MM2 (CD64^high^) phenotype is independent of age, race, sex, and treatment type and is characterized by a significant upregulation of CD64 and downregulation of CXCR2 and CD10 on mature neutrophils indicative of their lower maturation status. Additional components of the MM2 phenotype include upregulation of CD64 and activation markers on classical and patrolling monocytes, upregulation of PD-L1 on patrolling monocytes, and downregulation of cell signaling receptors on eosinophils. Functionally, neutrophil subpopulations from MM2 patients exhibit a higher capacity for the production of mitochondrial ROS. The findings presented here are consistent with the dysregulation of myelopoiesis resulting in the release of distinct myeloid cell subsets with altered phenotypic and functional properties (12, 20, 72–74).

The significance of assessment of neutrophil CD64 expression as a potential diagnostic tool is corroborated by previous observations demonstrating elevated STAT3 signaling and expression of CD64 on neutrophils from MGUS and MM patients and an association between upregulated CD64 and disease progression in MM patients treated with bortezomib, thalidomide, and dexamethasone (9, 75). In line with prior studies, the results presented here demonstrate that the presence of neutrophils with high CD64 expression in MM2 patients is significantly associated with the FLC ratio > 100 (**Table 1**). Consistent with a prior report, we did not observe an association between the level of CD64 expression on neutrophils and the presence of specific myeloma isotypes (9). The translational relevance of quantitative assessment of CD64 levels on neutrophils in clinical settings is supported by its use in other conditions, including the discrimination between sepsis and non-septic systemic inflammatory response syndrome (76, 77). In a human CD64 transgenic mouse model of B-cell lymphoma, CD64-directed bispecific antibodies were demonstrated to eliminate lymphoma cells and promote protection up to 100 days post tumor inoculation (78).

LDNs, commonly referred to as PMN-MDSCs in cancer, represent a pathologically activated neutrophil subpopulation expanded in various malignancies (13, 79). PMN-MDSCs are implicated in regulating the immune response in cancerous conditions and exert potent immunosuppressive properties including increased arginase-1 expression and inhibition of T-cell proliferation (15, 33, 34, 73, 74). Consistent with prior studies, we observe a significant increase in the frequencies of LDNs and imLDNs in MM2 patients relative to controls (**Figure 2B**). imN frequency detected in whole blood is significantly elevated in MM2 patients compared to MM1 or controls (**Figure 1A**). In concordance with our observations, prior studies reported the expansion of PMN-MDSC in MM and demonstrated their immunosuppressive capacity and ability to protect MM cells from chemotherapy-induced toxicity (28–34). Hsu et al. have shown that immature neutrophils exert enhanced migratory responses in breast-liver metastasis in response to complement 3a (11, 80). The shift in the immune cell compartments in the BME of MM results in an expansion of regulatory T cells (81) and MDSCs (28) promoting an immunosuppressive environment associated with altered function of myeloid cells consistent with the MM2 phenotype presented here (31, 33, 34). MM cells induce the development of MDSCs from healthy donor peripheral blood mononuclear cells *via* a bidirectional interaction between MM cells and MDSCs (29). Exosomes derived from the bone marrow stromal cells in MM patients alter myelopoiesis *via* activation of STAT1 and STAT3 pathways and increase of the levels of anti-apoptotic proteins Bcl-xL and Mcl-1 resulting in immature myeloid cells with immunosuppressive activity (72). The relevance of the determination of imN/PMN-MDSCs as an indicator of myeloid dysfunction and advanced disease is supported by prior reports demonstrating strong associations between PMN-MDSC frequencies and poor clinical outcomes in cancer (13, 79, 82).

A dysregulated functional capacity of myeloid cells in MM patients, including decreased capacity for phagocytosis and oxidative burst associated with elevated neutrophil CD64 levels, increased STAT3 phosphorylation, and immunosuppressive properties, has been previously reported (9). In the current study, we demonstrate an increase in the mitochondrial superoxide production in mature neutrophils of MM2 patients indicating a primed phenotype in the absence of signs of cellular activation or degranulation (**Figures 4A, B**). No differences in total ROS production were observed between the neutrophils of MM1 and MM2 patients (**Figures 4A, B**). Differences between the studies could be attributed to variances in the methods of assessment with prior studies utilizing different stimulants (e.g. fMLF), time of incubation, and other experimental conditions.

High serum levels of CXCL9 and CXCL10, closely related inflammatory cytokines interacting with chemokine receptor CXCR3, have been proposed as potential biomarkers of MM progression associated with poor overall survival (83, 84). High concentrations of CXCL9 and CXCL10 interfere with the infiltration of natural killer (NK) cells into the MM microenvironment and limit their anti-myeloma activity (85, 86). We demonstrate that CXCL9 is significantly elevated in MM2 patients and in patients with R-ISS stage III (**Figures 5** and **6**), consistent with prior studies (83, 84). We further show that the levels of CXCL9 closely correlate with the expression of CD64 on myeloid cells, in particular on classical and patrolling monocytes (R=0.8; *p*<0.0001; **Figure 5**). An elevated level of sCD163 has been proposed as an independent biomarker in MM with sCD163 levels in bone marrow aspirates and peripheral blood of MM patients shown to be associated with poor disease outcomes (66). Consistent with a prior report (66), we demonstrate that sCD163 levels are significantly elevated in the plasma of MM2 patients or patients diagnosed with R-ISS stage III disease (**Figure 5A** and **Figure 6D**).

Tumor progression, through immune editing of myeloid cells, has been demonstrated in experimental and clinical models of MM and patrolling monocytes were previously described as a potential indicator of circulating osteoclast precursors (33, 87). We did not observe a difference in the levels of classical and patrolling monocyte markers between the groups of patients with or without bone disease at the time of diagnosis (**Supplemental Figure 11**). The presented data provide a detailed characterization of circulating monocytic subsets, including the downregulation of CX3CR1 and upregulation of PD-L1 on patrolling monocytes in MM2 patients. To our knowledge, a detailed phenotypic characterization of circulating eosinophils has not been previously reported in patients with MM. It has been suggested that eosinophils can contribute to MM progression and demonstrate immunosuppressive properties (62, 88). Additionally, studies in allergic rhinitis (89) and pulmonary inflammation (90) demonstrate a critical role of CCR3 expression in the inflammatory pathway and recruitment of eosinophils. The data presented here demonstrating a significant downregulation of CCR3 on eosinophils in MM2 patients are consistent with prior studies and support active involvement of eosinophils in the pathogenesis of MM.

Limitations of this study include limited sample size and younger age of controls relative to MM patients. However, healthy controls did not contribute to the interpretation of findings from case-only analyses of patients in the MM1 and MM2 groups where no correlation with age was detected. Although MM phenotypes were primarily stratified based on neutrophil CD64 expression, other criteria including CXCR2 expression on mature neutrophils and CX3CR1 on monocytes were independently considered. Follow-up studies focusing on a detailed analysis of circulating innate immune cell subsets and their migratory properties present in the BME pre- and post-transplantation of treated and treatment-naïve patients would provide additional information regarding the evolving dynamics of myeloid cell populations in MM. Assessment of the immunosuppressive capacity of individual myeloid populations and their relationship to neoplastic plasma cells in the bone marrow would provide further insight into the biological and functional consequences of the dysregulated MM2 (CD64 ^high^) phenotype. In this study, we did not observe any statistically significant differences between MM1 and MM2 phenotypic features in relation to the treatment status. Longitudinal investigations with serial samples collected before, during, and after treatment are warranted to closely evaluate the predictive value of markers of myeloid dysregulation in MM and associated precancerous conditions.

The novel myeloid phenotype described here improves our understanding of the phenotypic and functional changes of neutrophils, monocytes, and eosinophils in MM patients. The results of this study highlight the importance of myeloid cell subsets as a parameter in the assessment and clinical monitoring of advanced-stage disease progression in MM and open new possibilities for the design of innovative therapeutic approaches.

## Data availability statement

The original contributions presented in the study are included in the article/**Supplementary Material**. Further inquiries can be directed to the corresponding author.

## Ethics statement

The studies involving human participants were reviewed and approved by Institutional Review Board of the University of Alabama at Birmingham. The patients/participants provided their written informed consent to participate in this study.

## Author contributions

Contribution: KO and ZH conceived, designed, and planned the experiments. KO, ZH, and EB coordinated the study, interpreted the data, and wrote the manuscript. KP and EB identified and recruited patients. EB, KP, and HC reviewed and provided clinical information for the study. KO, MD, AC, HP, and VK performed whole blood and PBMC processing for analysis of samples and contributed to antibody panel design. KO, MD, CF, and HP performed the ELISA studies and cytokine analyses. KO performed functional assays, Ki67 staining, and flow cytometry analysis. KO and EB analyzed clinical data and performed statistical analyses. All authors contributed to the article and approved the submitted version.

## Funding

This work was supported by the National Institutes of Health (R01HL129878 and R01DK108353, ZH; U01CA249955 and R01CA186646, EB). The content is solely the responsibility of the authors and does not necessarily represent the official views of the National Institutes of Health.
