# Supplementary material for: Distinct phenotype of neutrophil, monocyte, and eosinophil populations indicates altered myelopoiesis in a subset of patients with multiple myeloma
Source: Front Oncol. 2023 Jan 17;12:1074779. doi: 10.3389/fonc.2022.1074779 (PMC9888259; doi:10.3389/fonc.2022.1074779)
Supplement: Supplementary file 1 [file DataSheet_1.pdf]

Supplemental Table and Figures

Supplemental Table 1. Antibodies used for staining whole blood and PBMCs

Antibody	Source	Identifier
**Base panel**		
Anti-CD3-BV605™, clone OKT3	Biolegend	Cat# 317322; RRID: AB_2561991
Anti-CD14-APC/Fire™750, clone 63D3	Biolegend	Cat# 367120; RRID: AB_2572099
Anti-CD15-eF450, clone HI98	eBioscience™	Cat# 48-0159-42; RRID: AB_2016661
Anti-CD16-APC, clone eBioCB16	eBioscience™	Cat# 17-0168-42; RRID: AB_2016663
Anti-CD19-BV605™, clone HIB19	Biolegend	Cat# 302244; RRID: AB_2562015
Anti-CD62L-BV711, clone DREG-56	BD Biosciences	Cat# 740783; RRID: AB_2740446
Anti-CD193 (CCR3)-BV510, clone 5E8	BD Biosciences	Cat# 563071; RRID: AB_2737988
**Analysis of whole blood and PBMCs**		
Anti-CD10-Per CP/Cy5.5, clone HI10a	Biolegend	Cat# 312216; RRID: AB_10642819
Anti-CD11b-PerCP/Cy5.5, clone ICRF44	Biolegend	Cat# 301328; RRID: AB_10933428
Anti-CD31-FITC, clone WM59	Biolegend	Cat# 303104; RRID: AB_314330
Anti-CD36-FITC, clone 5-271	Biolegend	Cat# 336204; RRID: AB_1575025
Anti-CD54-FITC, clone HA58	Biolegend	Cat# 353108; RRID: AB_10900254
Anti-CD63-PerCP/Cy5.5, clone H5C6	Biolegend	Cat# 353020; RRID: AB_2561685
Anti-CD64-PE/Cy7, clone 10.1	Biolegend	Cat# 305022; RRID: AB_2561584
Anti-CD66b-FITC, clone G10F5	Biolegend	Cat# 305104; RRID: AB_314496
Anti-CD142-PE, clone NY2	Biolegend	Cat# 365204; RRID: AB_2564566
Anti-CD163-PE/Cy7, clone GHI/61	Biolegend	Cat# 333614; RRID: AB_2562641
Anti-CD169-PE, clone 7-239	Biolegend	Cat# 346004; RRID: AB_2189029
Anti-CD274 (PD-L1)-PE/Cy7, clone 29E.2A3	Biolegend	Cat# 329718; RRID: AB_2561687
Anti-CD284 (TLR-4)-PE/Cy7, clone HTA125	eBioscience™	Cat# 25-9917-42; RRID: AB_11219074
Anti-CD182 (CXCR2)-PerCP/Cy5.5, clone 5E8/CXCR2	Biolegend	Cat# 320718; RRID: AB_2564599
Anti-CD184 (CXCR4)-PE, clone 12G5	Biolegend	Cat# 306506; RRID: AB_314612
Anti-CX3CR1-PerCP/Cy5.5, clone 2A9-1	Biolegend	Cat# 341614; RRID: AB_11219203
Anti-HLA-DR-PE/Cy7, clone L243	Biolegend	Cat# 307616; RRID: AB_493588
Anti-LOX-1-PE, clone 15C4	Biolegend	Cat# 358604; RRID: AB_2562181
Anti-MPO-PE, clone 2C7	Novus	Cat# NB100-64803; RRID: AB_964678
**Absolute cell count panel**		
Anti-CD3-FITC, clone OKT3	Biolegend	Cat# 317306; RRID: AB_571907
Anti-CD4-PE, clone RPA-T4	Biolegend	Cat# 300508; RRID: AB_314076
Anti-CD8a-PerCP/Cy5.5, clone RPA-T8	Biolegend	Cat# 301032; RRID: AB_2288911
Anti-CD14-APC/Fire™750, clone 63D3	Biolegend	Cat# 367120; RRID: AB_2572099

Anti-CD15-eF450, clone HI98	eBioscience™	Cat# 48-0159-42; RRID: AB_2016661
Anti-CD16-APC, clone eBioCB16	eBioscience™	Cat# 17-0168-42; RRID: AB_2016663
**Ki67 panel**		
Anti-CD3-BV605™, clone OKT3	Biolegend	Cat# 317322; RRID: AB_2561991
Anti-CD14-APC/Fire™750, clone 63D3	Biolegend	Cat# 367120; RRID: AB_2572099
Anti-CD15-eF450, clone HI98	eBioscience™	Cat# 48-0159-42; RRID: AB_2016661
Anti-CD16-PE, clone eBioCB16	eBioscience™	Cat# 12-0168-42; RRID:AB_11043436
Anti-CD19-BV605™, clone HIB19	Biolegend	Cat# 302244; RRID: AB_2562015
Anti-CD62L-BV711, clone DREG-56	BD Biosciences	Cat# 740783; RRID: AB_2740446
Anti-CD64-PE/Cy7, clone 10.1	Biolegend	Cat# 305022; RRID: AB_2561584
Anti-CD182 (CXCR2)-PerCP/Cy5.5, clone 5E8/CXCR2	Biolegend	Cat# 320718; RRID: AB_2564599
Anti-CD193 (CCR3)-BV510, clone 5E8	BD Biosciences	Cat# 563071; RRID: AB_2737988
Anti-Ki67-APC, clone Ki67	Biolegend	Cat# 350514; RRID:AB_10959327
**DCFDA (ROS) panel**		
Anti-CD14-APC/Fire™750, clone 63D3	Biolegend	Cat# 367120; RRID: AB_2572099
Anti-CD15-eF450, clone HI98	eBioscience™	Cat# 48-0159-42; RRID: AB_2016661
Anti-CD16-PE, clone eBioCB16	eBioscience™	Cat# 12-0168-42; RRID:AB_11043436
Anti-CD64-PE/Cy7, clone 10.1	Biolegend	Cat# 305022; RRID: AB_2561584
Anti-CD101-PE, clone BB27	Biolegend	Cat# 331012; RRID:AB_2716107
Anti-CD193 (CCR3)-BV510, clone 5E8	BD Biosciences	Cat# 563071; RRID: AB_2737988
**MitoSOX, TMRE, MitoTracker Green panel**		
Anti-CD14-APC/Fire™750, clone 63D3	Biolegend	Cat# 367120; RRID: AB_2572099
Anti-CD15-eF450, clone HI98	eBioscience™	Cat# 48-0159-42; RRID: AB_2016661
Anti-CD16-PE, clone eBioCB16	eBioscience™	Cat# 12-0168-42; RRID:AB_11043436
Anti-CD64-PE/Cy7, clone 10.1	Biolegend	Cat# 305022; RRID: AB_2561584
Anti-CD193 (CCR3)-BV510, clone 5E8	BD Biosciences	Cat# 563071; RRID: AB_2737988
**pHrodo panel**		
Anti-CD64-PE/Cy7, clone 10.1	Biolegend	Cat# 305022; RRID: AB_2561584
Anti-CD66b-FITC, clone G10F5	Biolegend	Cat# 305104; RRID: AB_314496
Anti-CD182 (CXCR2)-PerCP/Cy5.5, clone 5E8/CXCR2	Biolegend	Cat# 320718; RRID: AB_2564599

Figure legends

Figure S1. Gating strategies for innate immune cell subpopulations.

(A) Gating strategy for the identification of monocyte, neutrophil, and eosinophil subpopulations. All cells were gated as single cells with a negative gate to remove T and B lymphocytes with lineage markers CD3 and CD19, respectively. Monocytes were gated following the removal of $CD15^{+}$ cells. SSC/FSC low events were removed to ensure the exclusion of any potential debris (not shown). Classical monocytes (Cl Mo) were identified as $CD14^{high}CD16^{low}$ and patrolling monocytes (Pt Mo) were identified as $CD14^{low}CD16^{high}$. Neutrophils were gated following the removal of eosinophils and monocytes utilizing CCR3 and CD14, respectively. SSC/FSC low events were then excluded (not shown) with neutrophils identified as $SSC^{high}CD15^{+}$ cells with mature neutrophils (mNs) identified as $CD15^{+}CD16^{+}$ and immature neutrophils (imNs) identified as $CD15^{+}CD16^{-}$. Eosinophils (Eos) were gated following the removal of $CD16^{+}$ cells and SSC/FSC low events (not shown) and were identified as $CCR3^{+}CD15^{+}CD16^{-}$. **(B)** Ki67 gating of neutrophil subpopulations. All cells were gated using a previously determined FMO gate for the APC fluorochrome. Cells positive for Ki67 were determined by applying the FMO gate to the Ki67-stained cells; FMO = fluorescent minus one.

Figure S2. Analysis of neutrophil phenotype in MM patients.

(A) Expression of surface markers of neutrophil activation, cell signaling, and migration of healthy donors (HD) (n=15) and multiple myeloma (MM) patients (n=35). **(B)** Percentage of $CD64^{neg}$ mNs in HD, MM1, and MM2 patients. $CD64^{neg}$ cells were determined as events below the gate set at 95% of events of fluorescence minus one (FMO) control from a healthy donor. MFI, median fluorescent intensity. Statistical analyses were performed using the Mann-Whitney rank-sum test with p values indicated. Bars represent median values.

Figure S3. Analysis of the phenotype of neutrophil subpopulations and myeloid cell ratios in MM patients.

(A) Expression of surface markers of neutrophil activation, cell signaling, and migration on mNs. **(B)** Frequency of total and mature neutrophils per ml of blood and neutrophil to T-lymphocyte ratio. **(C)** Frequency of M-MDSC (monocytic myeloid-derived suppressor cells) per ml of blood and T-lymphocyte to monocyte ratio. **(D)** Percentage of $Ki67^{+}$ mNs of total mNs. **(E)** Expression of neutrophil surface markers on imNs. MFI, median fluorescent intensity; NLR, neutrophil to T: lymphocyte ratio; LMR, T: lymphocyte to monocyte ratio. Statistical analyses were performed using the Mann-Whitney rank-sum test with p values are indicated; bars and lines represent median values and simple linear regression analysis, respectively.

Figure S4. Analysis of monocyte phenotypes in MM patients.

(A-F) Surface levels of monocyte maturation, activation, cell signaling and migration markers on classical **(A-C)** and patrolling **(D-F)** monocytes. MFI, median fluorescent intensity. Statistical analyses were performed using the Mann-Whitney rank-sum test with p values indicated. Bars represent median values.

Figure S5. Analysis of the eosinophil phenotype in MM patients.

Surface levels of maturation, lineage, activation, cell signaling, and migration markers on eosinophils. MFI, median fluorescent intensity. Statistical analyses were performed using the Mann-Whitney rank-sum test with *p* values indicated. Bars represent median values.

Figure S6. Phagocytic capacity of myeloid subpopulations.

(A-B) Phagocytic capacity was detected by phagocytosis of pHrodo^{TM} Red *E. coli* BioParticles^{TM} on neutrophil **(A)** and monocyte **(B)** subpopulations. MFI, median fluorescent intensity. Statistical analyses were performed using the Mann-Whitney rank-sum test with *p* values indicated. Bars represent median values.

Figure S7. Plasma levels of markers of inflammation in relation to MM phenotypes.

Levels of cytokines and markers of myeloid activation in plasma of HD and MM1 and MM2 patients. Statistical analyses were performed using the Mann-Whitney rank-sum test with *p* values indicated. Bars represent median values.

Figure S8. Analysis of the potential effect of autologous stem cell transplant on MM2 myeloid phenotype components.

(A) Correlation between the surface levels of characteristic markers of the MM2 phenotype on mature neutrophils, classical and patrolling monocytes, and eosinophils with the time period between autologous hematopoietic stem cell transplant (aHSCT) and blood draw (BD). **(B)** Surface levels of myeloid subpopulations separated based on aHSCT status. $aHSCT_{prior}$ is indicative of aHSCT performed > 100 days prior to BD; $aHSCT_{after}$ is indicative of aHSCT performed after BD. MFI = median fluorescent intensity. Statistical analyses were performed using the Spearman correlation test **(A)** or the Mann-Whitney ranked-sum test **(B)**. Spearman correlation coefficients R and *p* values are indicated; bars and lines represent median values and simple linear regression analysis, respectively.

Figure S9. Analysis of the potential effect of induction therapy on MM2 myeloid phenotype components.

Correlation between the time period between induction therapy (IT) and BD and the surface levels of characteristic markers of the MM2 phenotype on mature neutrophils, classical and patrolling monocytes, and eosinophils. MFI = median fluorescent intensity. Statistical analyses were performed using the Spearman correlation test. Spearman correlation coefficients R and *p* values are indicated; bars and lines represent median values and simple linear regression analysis, respectively.

Figure S10. Analysis of the relationship between the characteristic features of the MM2 myeloid phenotype and MM immunoglobulin isotype.

(A-B) Surface levels of characteristic markers of the MM2 phenotype on mature neutrophils, classical and patrolling monocytes, and eosinophils separated by free light chain **(A)** or immunoglobulin **(B)** isotype. MFI = median fluorescent intensity. Statistical analyses performed with the Mann-Whitney ranked-sum test with *p* values indicated. Bars represent median values.

Figure S11. Analysis of the relationship between the characteristic features of the MM2 myeloid phenotype and the presence of bone disease.

Surface levels of characteristic markers of the MM2 phenotype on mature neutrophils, classical monocytes, patrolling monocytes, and eosinophils separated by presence of bone disease. MFI = median fluorescent intensity. Statistical analyses performed with the Mann-Whitney ranked-sum test with *p* values indicated. Bars represent median values.

Figure S12. Characteristic features of the MM2 phenotype in relation to the R-ISS staging.

(A-C) Expression of surface markers on mature neutrophils **(A)**, classical monocytes **(B)**, and patrolling monocytes **(C)** stratified using the R-ISS staging system. MFI, median fluorescent intensity; statistical analyses were performed using the Kruskal-Wallis one way ANOVA test followed by Dunn's post hoc test with *p* values indicated. Bars represent median values.

Figure S13. Characteristic features of the MM2 phenotype in relation to the Durie-Salmon staging.

(A-C) Surface levels of the characteristic markers of the MM2 phenotype on mature neutrophils, **(A)** classical monocytes **(B)**, and patrolling monocytes **(C)** stratified using the DS staging system. **(D)** Levels of cytokines and myeloid markers of activation stratified using the DS staging system. MFI, median fluorescent intensity. Statistical analyses were performed using the Kruskal-Wallis one-way ANOVA test followed by Dunn's post hoc test with *p* values as indicated. Bars represent median values.

Figure S14. Neutrophil characteristic and functional features of the MM2 phenotype.

(A) Representative cytograms and histogram overlays of neutrophil markers of mNs of HD and MM1 and MM2 patients. **(B-C)** Representative cytograms of mLDNs of HD and MM1 and MM2 patients and histogram overlays of MitoSOX **(B)** and pHrodo **(C)**. Mitochondrial superoxide production by MitoSOX^{TM} Red shown with stimulation of LDNs with PMA. Phagocytic capacity was detected by phagocytosis of pHrodo^{TM} Red *E. coli* BioParticles^{TM} by LDNs. **(D)** Representative histogram overlays of antibody isotype control staining of neutrophils from HD, MM1, and MM2 patients.

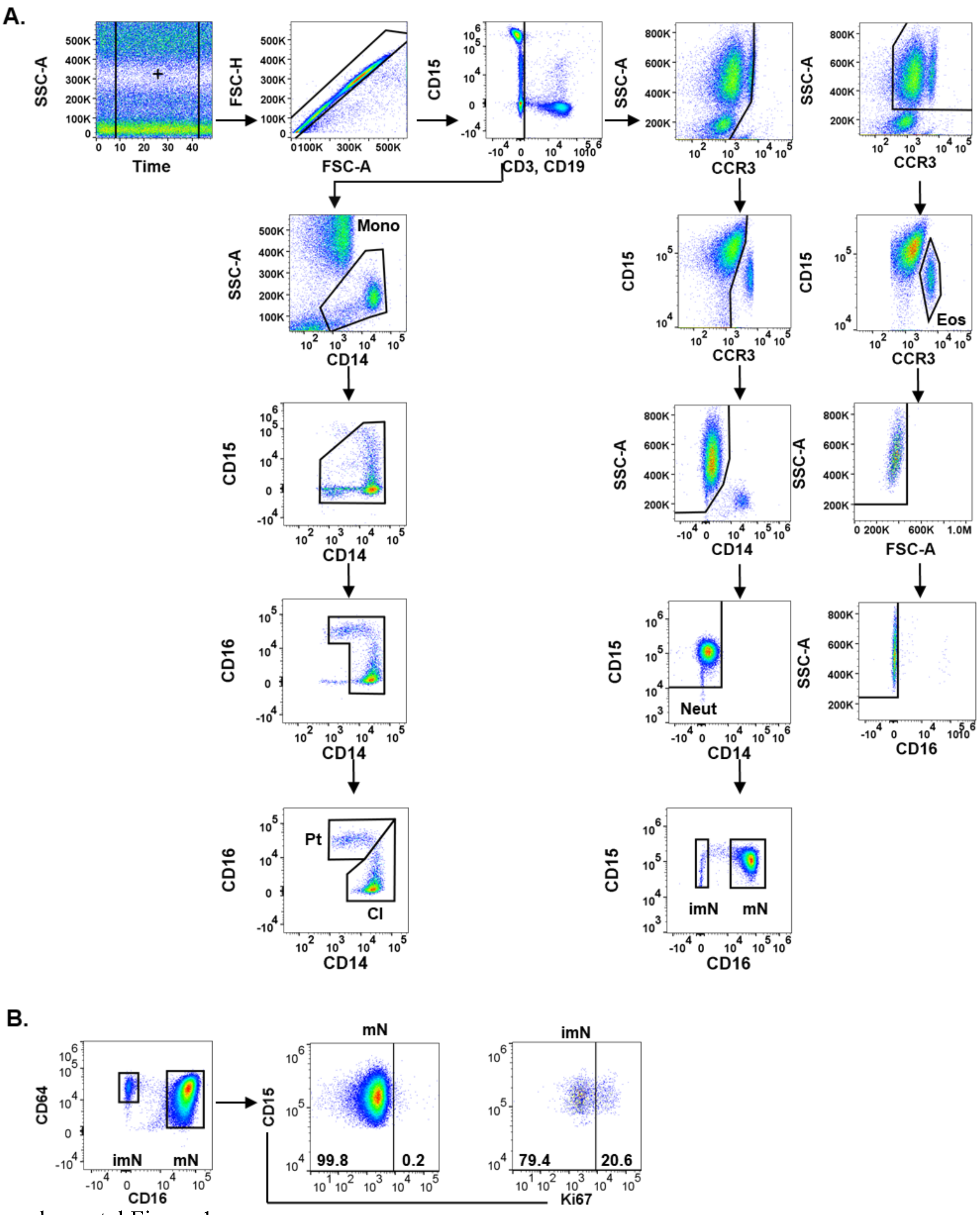

Supplemental Figure 1

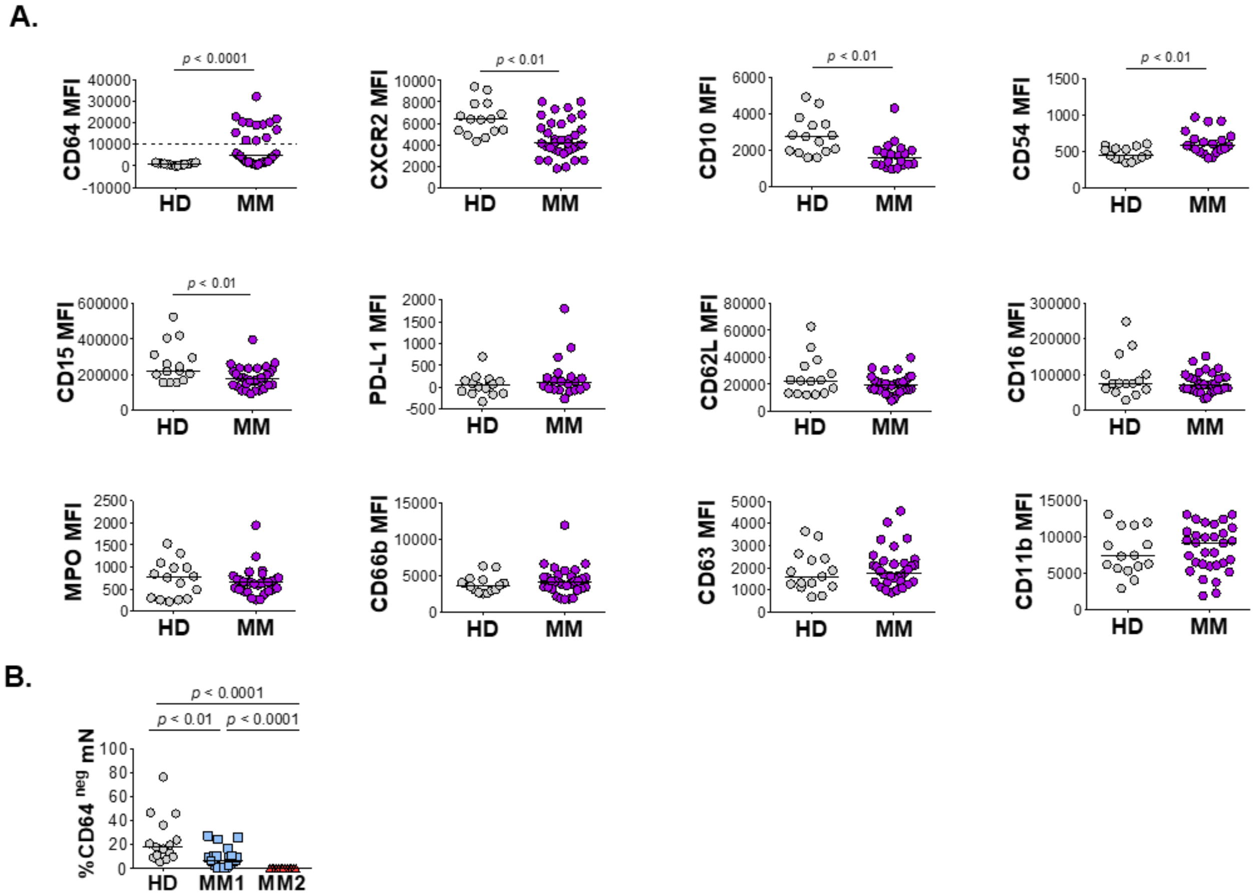

Supplemental Figure 2

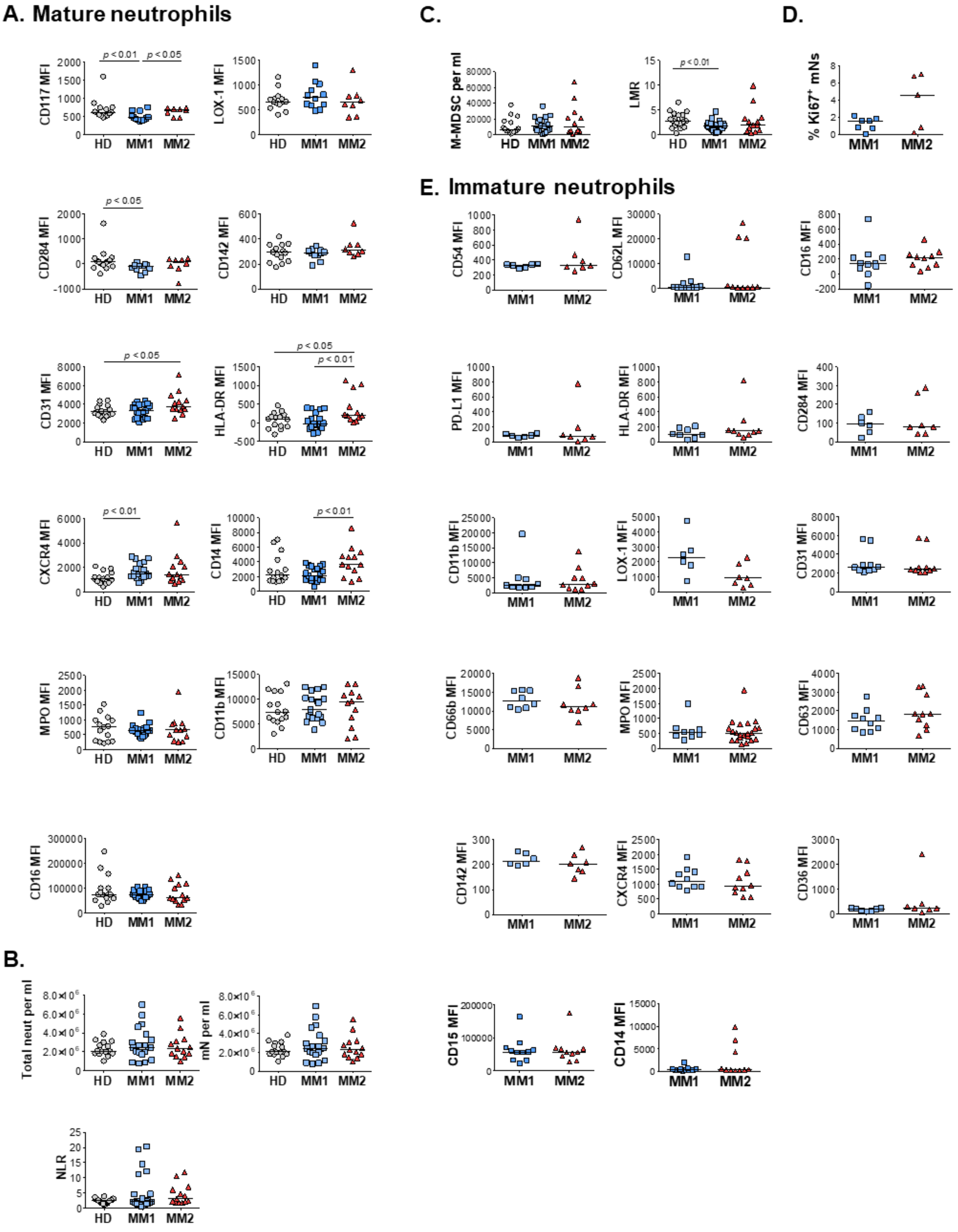

Supplemental Figure 3

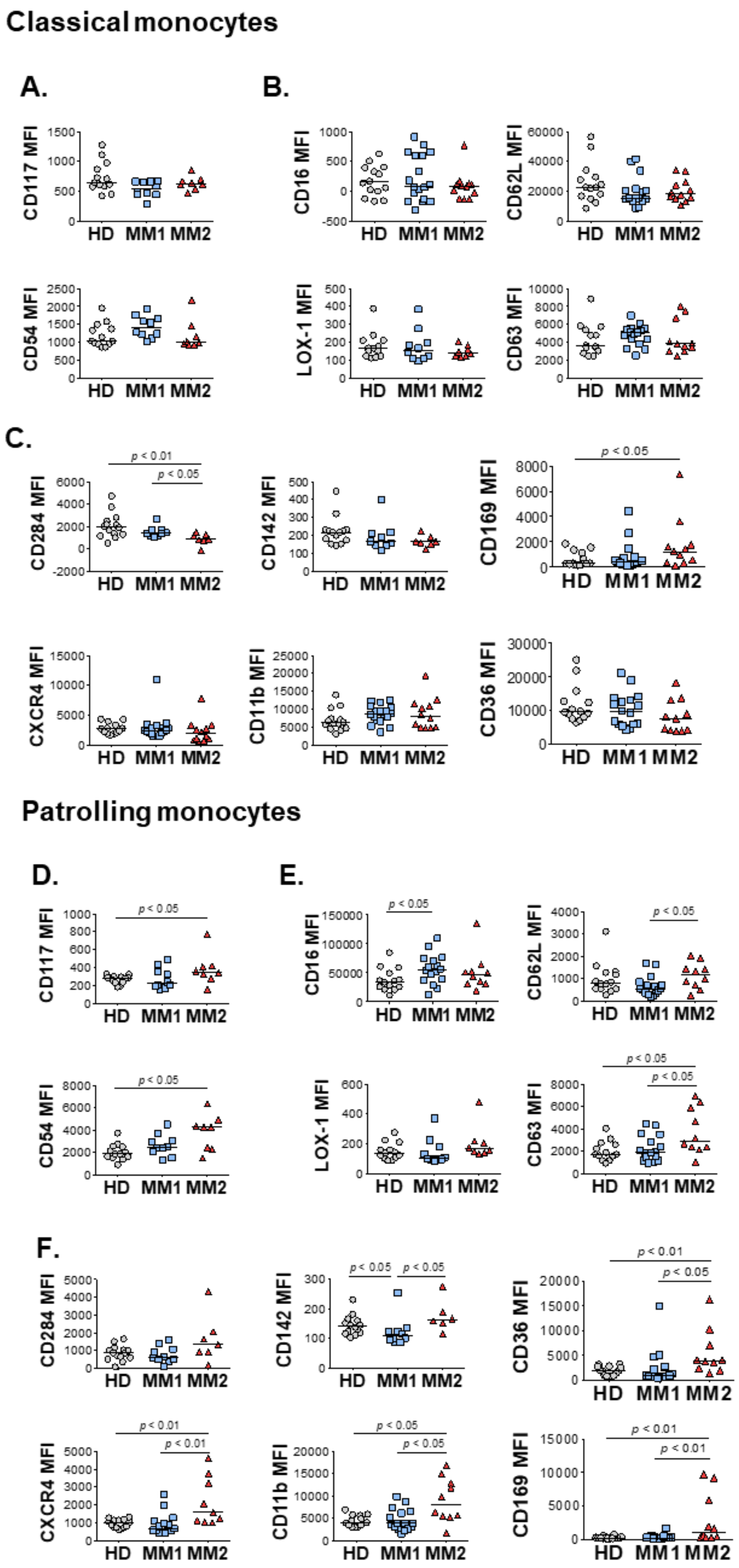

Supplemental Figure 4

Eosinophils

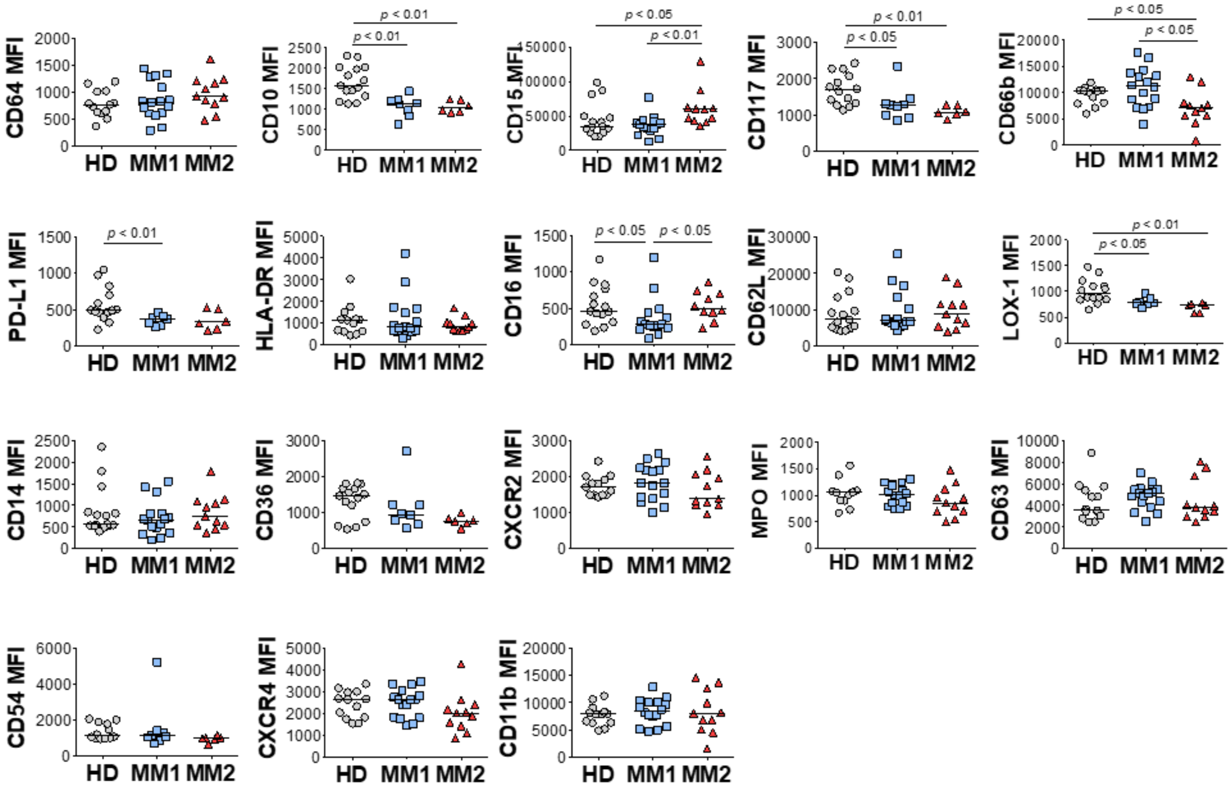

Supplemental Figure 5

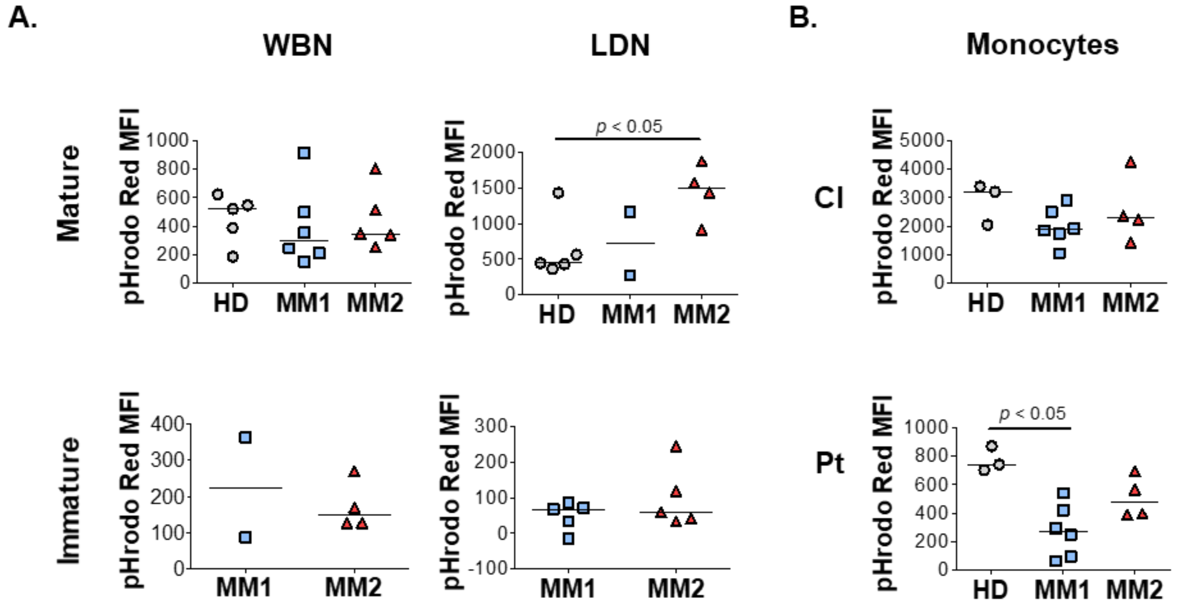

Supplemental Figure 6

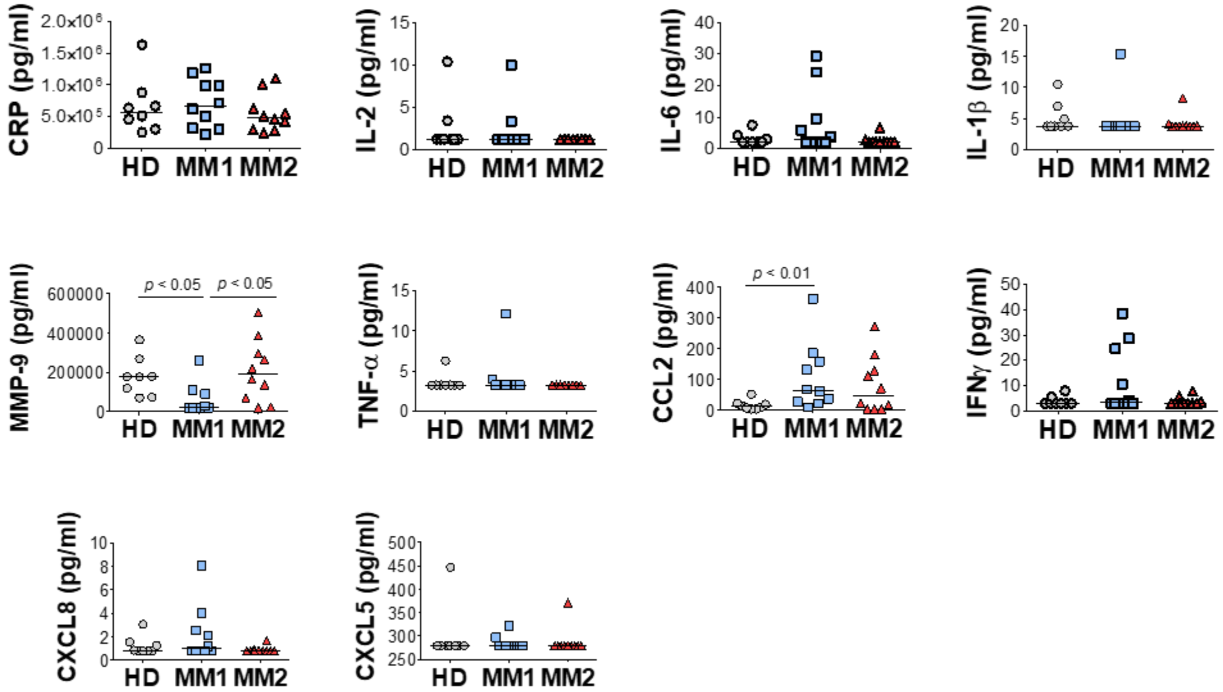

Supplemental Figure 7

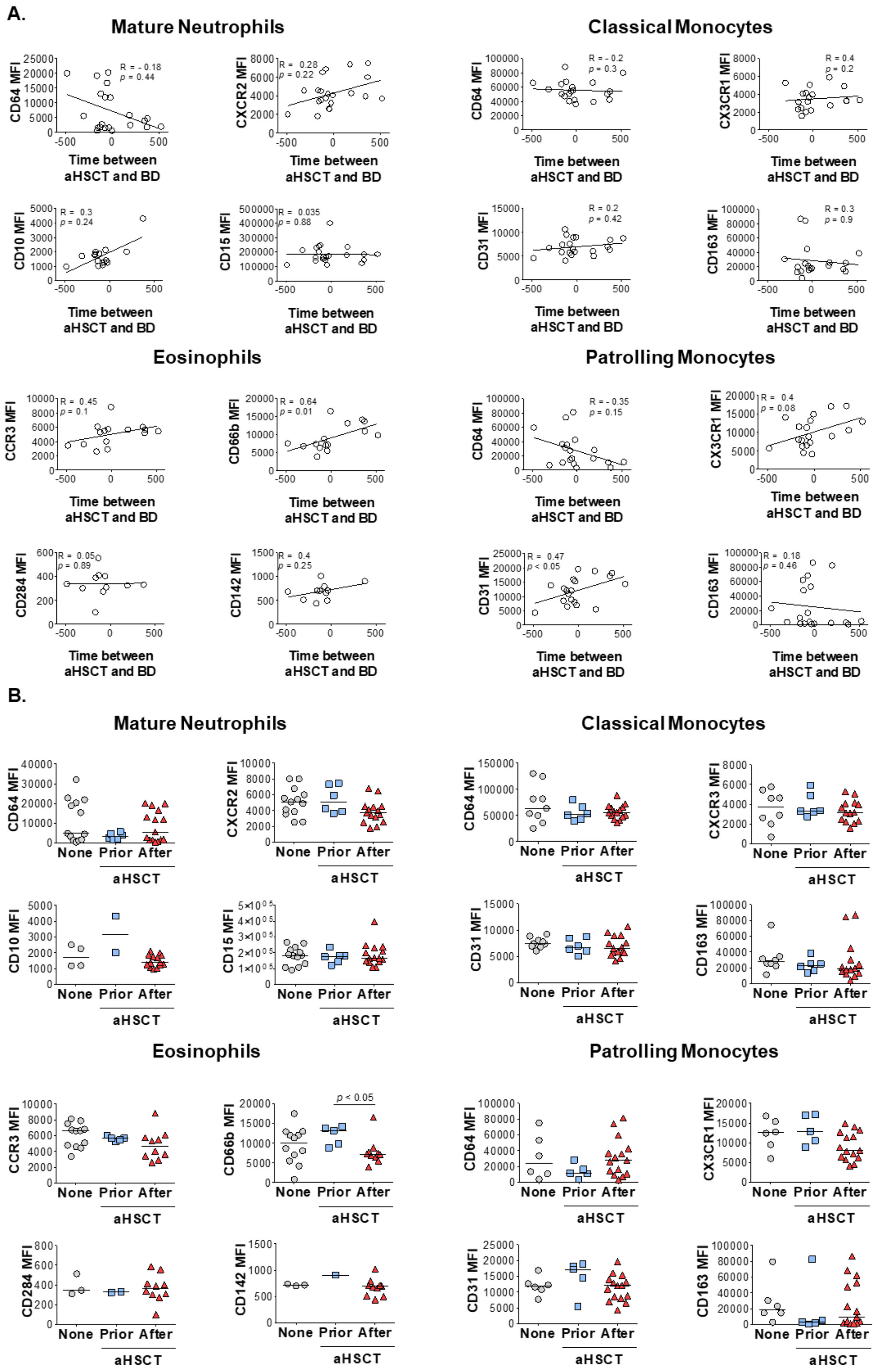

Supplemental Figure 8

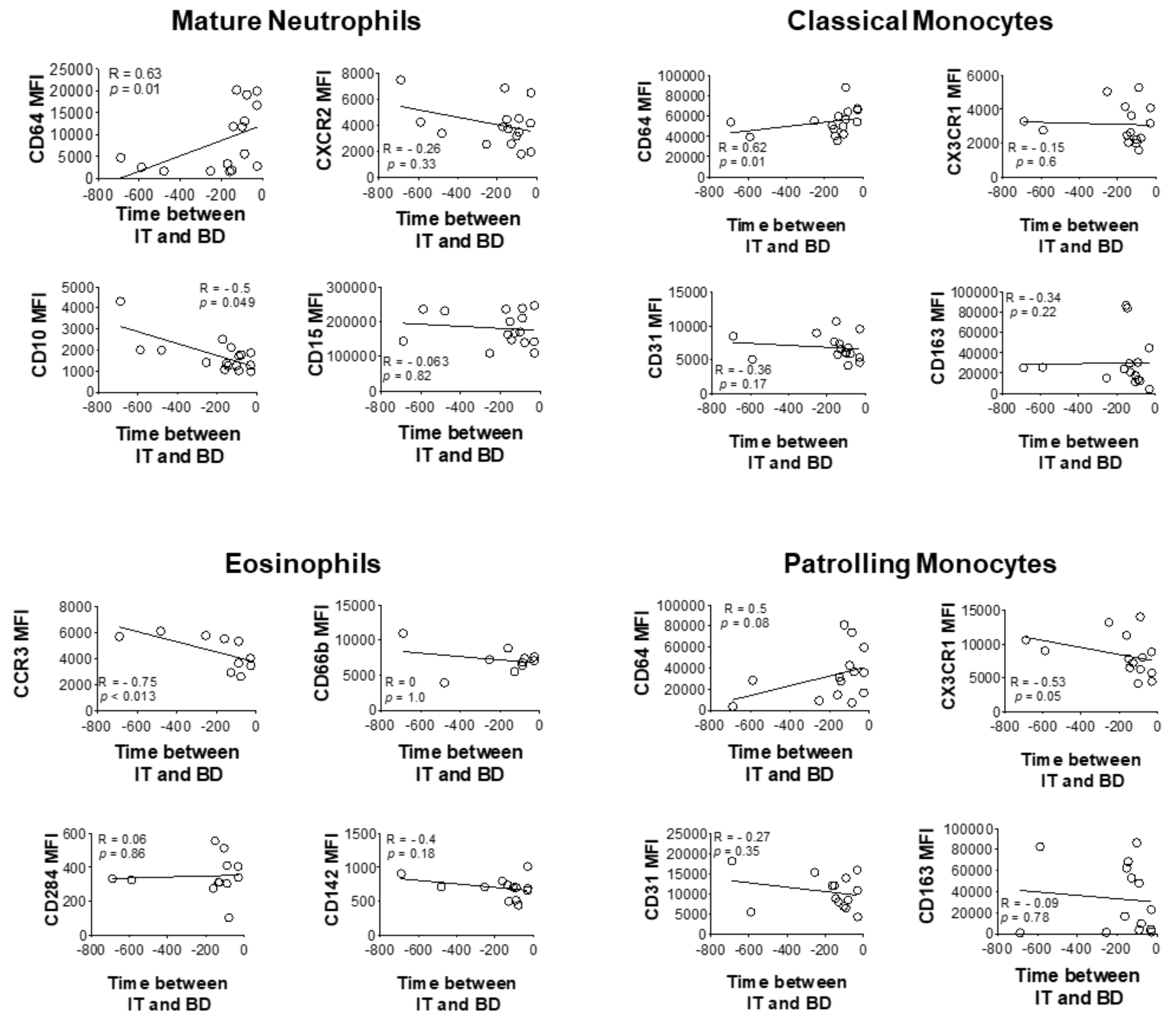

Supplemental Figure 9

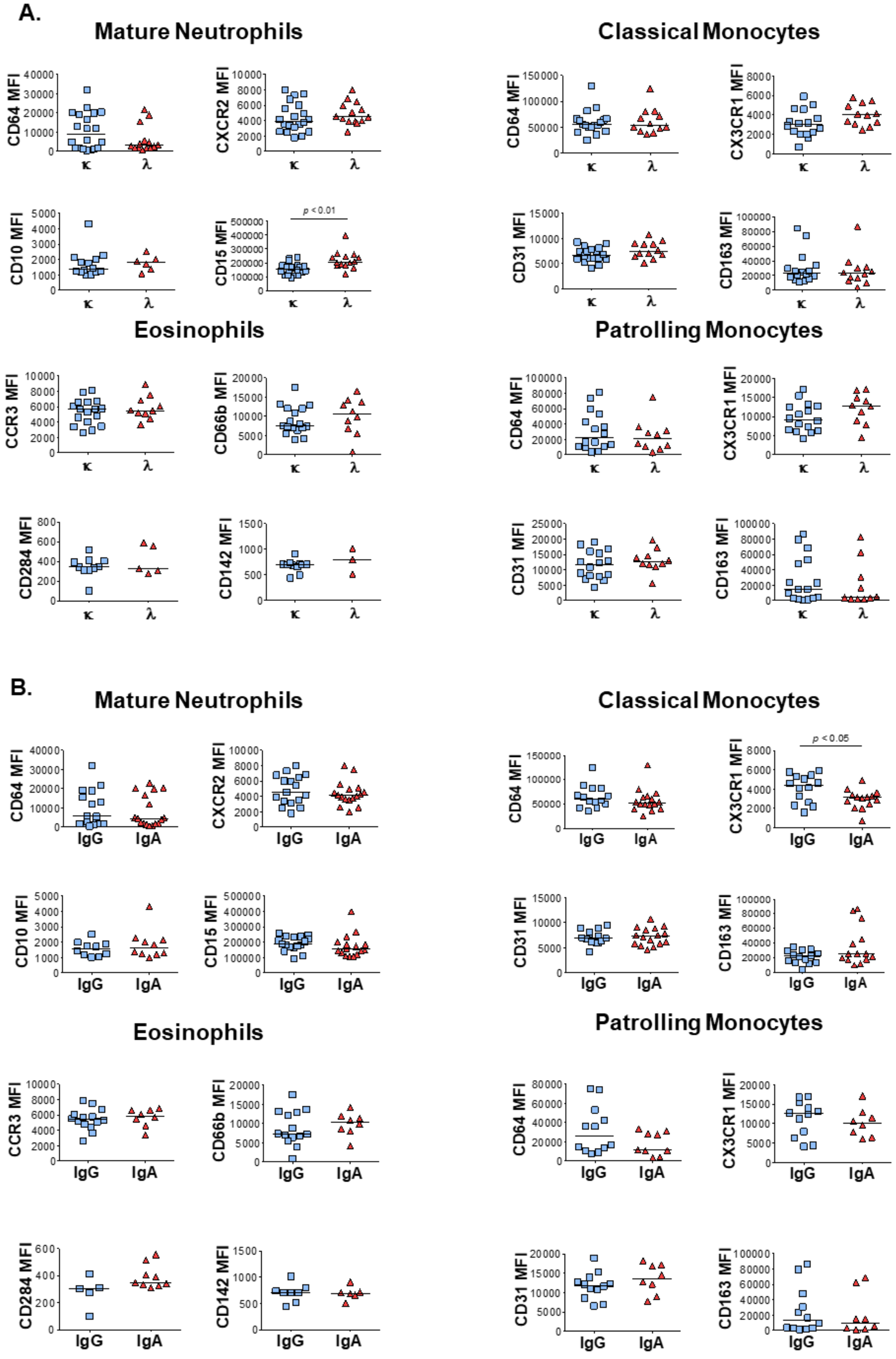

Supplemental Figure 10

1. Seven MM patients were light-chain restricted and not included in the analyses for Supplemental Figure 10A.

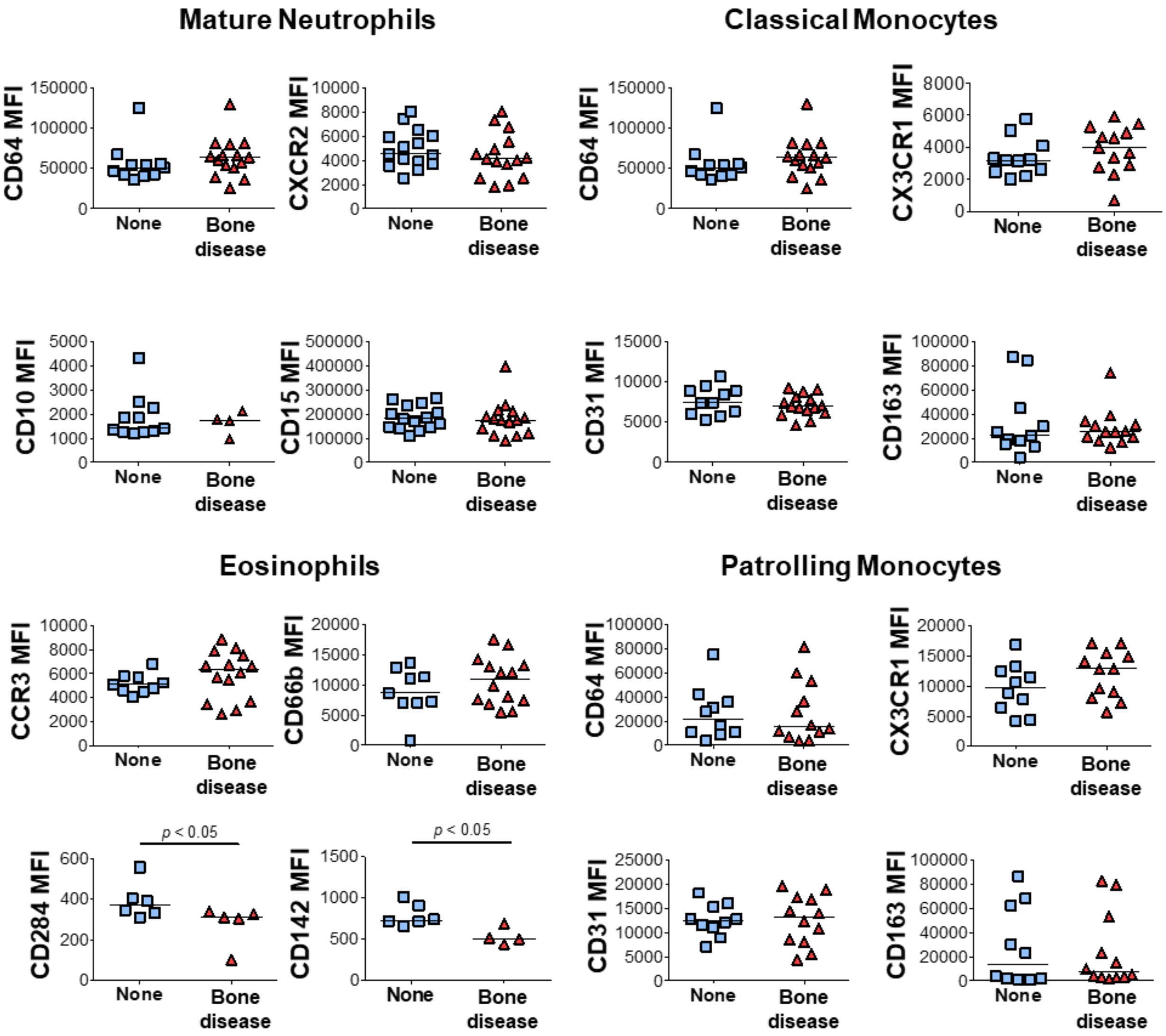

Supplemental Figure 11

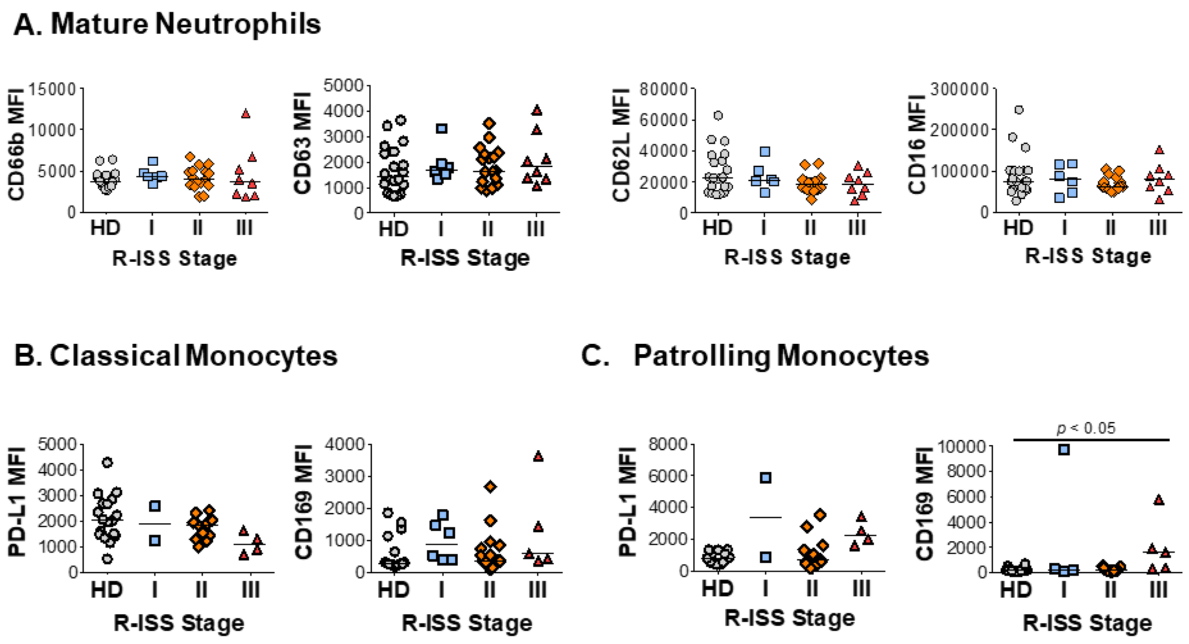

Supplemental Figure 12

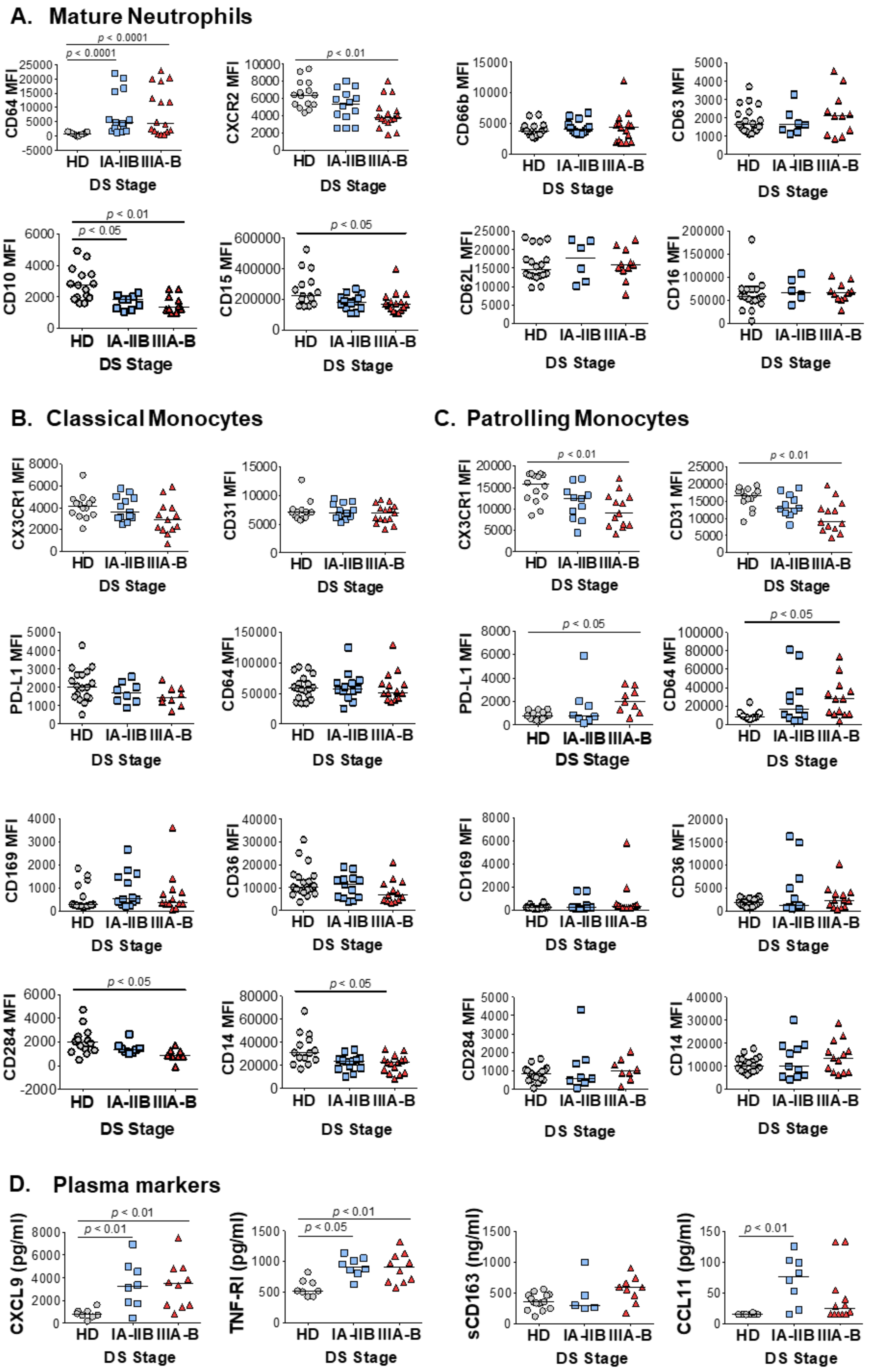

Supplemental Figure 13

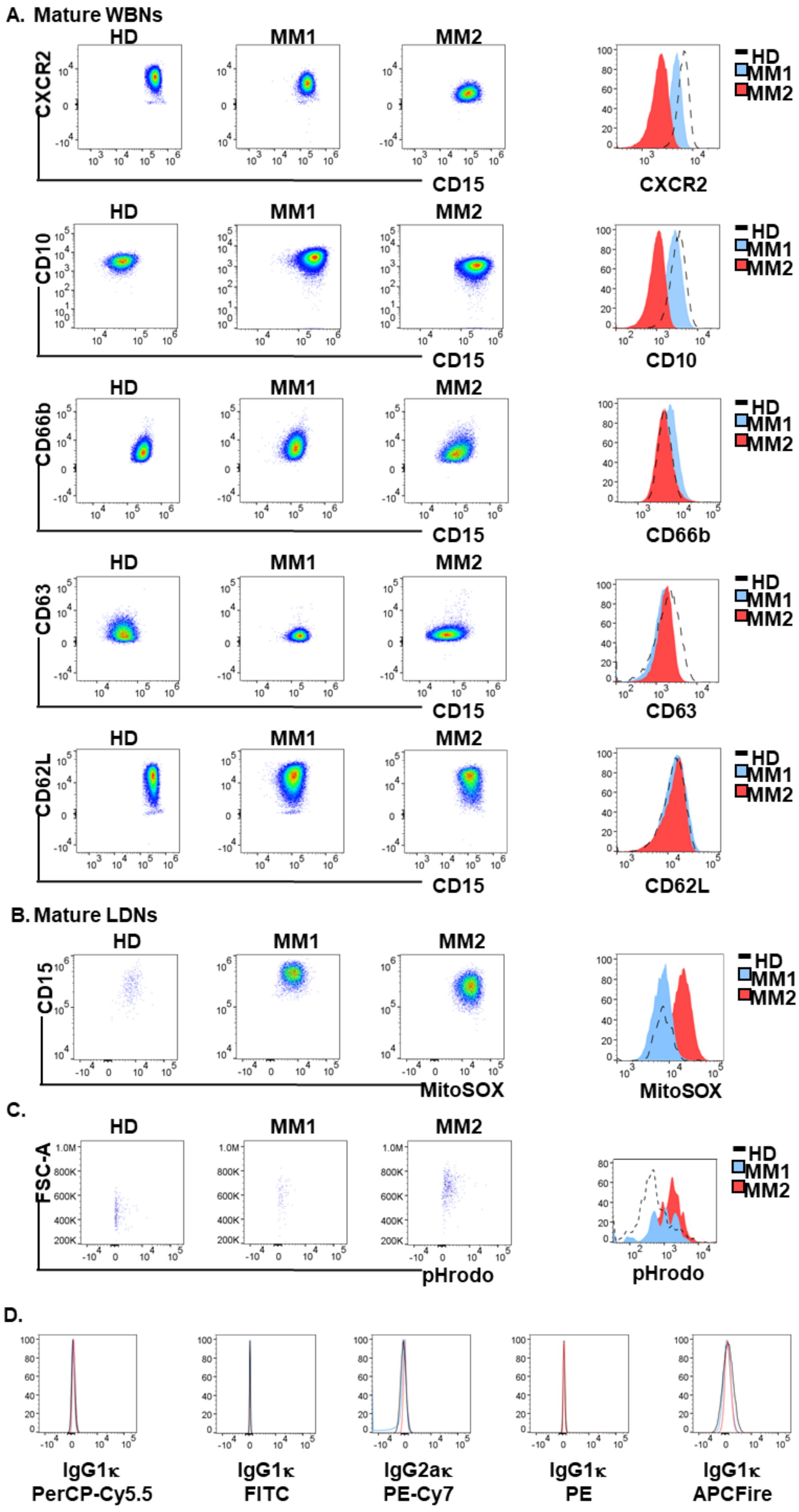

Supplemental Figure 14
